# CADM1 impairs the effect of miR-1246 on promoting cell cycle progression in chemo-resistant leukemia cells

**DOI:** 10.1186/s12885-023-11458-1

**Published:** 2023-10-09

**Authors:** Bei Xie, Lei Zhao, Zhewen Zhang, Cunmin Zhou, Ye Tian, Yingying Kang, Jing Chen, Hulai Wei, Linjing Li

**Affiliations:** 1 Department of Immunology, School of Basic Medical Sciences, Lanzhou University, No. 199 Donggang West Road, Lanzhou, 730000 Gansu China; 2 Shaanxi Meili Omni-Honesty Animal Health Co., Ltd, Xi’an, 710000 Shaanxi China; 3 The First Hospital of Lanzhou University, Lanzhou, 730000 Gansu China; 4 The Second Hospital of Lanzhou University, Lanzhou, 730000 Gansu China

**Keywords:** Chemo-resistant leukemia cells, Cell cycle, CADM1, miR-1246, CDKs/Cyclins axis

## Abstract

The interruption of normal cell cycle execution acts as an important part to the development of leukemia. It was reported that microRNAs (miRNAs) were closely related to tumorigenesis and progression, and their aberrant expression had been demonstrated to play a crucial role in numerous types of cancer. Our previous study showed that miR-1246 was preferentially overexpressed in chemo-resistant leukemia cell lines, and participated in process of cell cycle progression and multidrug resistant regulation. However, the underlying mechanism remains unclear. In present study, bioinformatics prediction and dual luciferase reporter assay indicated that CADM1 was a direct target of miR-1246. Evidently decreased expression of CADM1 was observed in relapsed primary leukemia patients and chemo-resistant cell lines. Our results furtherly proved that inhibition of miR-1246 could significantly enhance drug sensitivity to Adriamycin (ADM), induce cell cycle arrest at G0/G1 phase, promote cell apoptosis, and relieve its suppression on CADM1 in K562/ADM and HL-60/RS cells. Interference with CADM1 could reduce the increased drug sensitivity induced by miR-1246 inhibition, and notably restore drug resistance by promoting cell cycle progression and cell survival via regulating CDKs/Cyclins complexes in chemo-resistant leukemia cells. Above all, our results demonstrated that CADM1 attenuated the role of miR-1246 in promoting cell cycle progression and cell survival, thus influencing multidrug resistance within chemo-resistant leukemia cells via CDKs/Cyclins. Higher expression of miR-1246 and lower expression of CADM1 might be risk factors for leukemia.

**Supplementary Information:**

The online version contains supplementary material available at 10.1186/s12885-023-11458-1.

## Introduction

Leukemia is the commonest childhood cancer, which arises from clonal proliferation of abnormal hematopoietic cells, leading to the interruption of normal marrow function. Various clinical manifestations of leukemia stem from irregular proliferation of the malignant clones and bone marrow failure [1]. Numerous studies indicate that microRNAs (miRNAs) play vital roles in a variety of biological processes associated with cancer, such as cell proliferation, metastasis, cell cycle distribution, apoptosis and drug sensitivity [2, 3].

miRNA is known as one class of small non-coding regulatory RNAs with sizes of 17 ~ 25 nucleotides that post-transcriptionally regulate gene expression by recognizing complementary target sites in the 3’ untranslated region (UTR) of target mRNAs [4]. Studies reported that dysregulated miRNAs was associated with the initiation and progression of leukemia [5]. Nie et al. declared that dramatically decreased miR-204 was found in serum samples and cells of acute myeloid leukemia (AML), and its overexpression would repress cell proliferation, migration, invasion, and induce cell apoptosis by targeting Hepatocyte growth factor (HGF)/c-Met pathway [6]. Zhang et al. demonstrated that interference with miR-18a could suppress the proliferative potential and activate apoptotic process by elevating the expression of PTEN mRNA in WEHI-3 and THP-1 leukemia cells [7]. Wu et al. reported that inhibition of miR-362-5p expression could significantly restrain cell proliferation, induce G0/G1 phase arrest and attenuate tumor growth in AML by targeting growth arrest-specific 7 (GAS7) [8].

Few studies had focused on miR-1246 which acted as a promotor in leukemia. Chen et al. uncovered that miR-1246, highly-expressed in AML cell-derived extracellular vesicles (EVs), could improve the survival abilities of leukemia stem cells by activating LRIG1-mediated STAT3 pathway [9]. Our previous study proved that the expression of miR-1246 was significantly increased in chemo-resistant leukemia cells. Inhibition of miR-1246 could negatively regulate AXIN2 and GSK-3β thus inactivate Wnt/β-catenin pathway, and suppress the expression of P-glycoprotein (P-gp) then enhance the drug sensitivity of chemo-resistant leukemia cells [2]. Besides that, the cell cycle distribution altered along with the expression level of miR-1246. However, the underlying mechanisms remain unclear. Here, we investigated the potential role of miR-1246 on cell cycle progression, drug resistance and apoptosis, providing more evidences to confirm the effect of miR-1246 on leukemia, especially chemo-resistant leukemia. In addition, our study attempts to offer more therapeutic targets and diagnostic markers for leukemia.

## Materials and methods

### Cell culture and treatment

The human leukemia cell lines K562, HL-60 and K562/ADM cells were purchased from Institute of Hematology of Chinese Academy of Medical Sciences (Tianjin, China). HL-60/RS cell, as a stable Adriamycin (ADM) resistant cell line, was generated from HL-60 cells by ADM-selection in our laboratory [10]. All cell lines were maintained in RPMI-1640 culture medium (Gibco, Paisley, UK) supplemented with 10% fetal bovine serum (FBS; Hyclone, Logan, UT, USA), 100 U/mL penicillin, 100 µg/mL streptomycin, and incubated in humidified 5% CO_2_ atmosphere at 37℃. The cells in the logarithmic phase were used for subsequent experiments.

### Collection of clinical specimens

Blood samples from patients who were newly diagnosed with leukemia and then acquired partial or complete relapse (n = 14) were recruited for this study. Matching blood samples from donors with no history of leukemia were included. All the blood samples were collected from the Hematology Department of the First Hospital of Lanzhou University between March, 2017 and March, 2020. Written informed consents were obtained from each participant. This study was in accordance with The Code of Ethics of the World Medical Association (Declaration of Helsinki) for experiments involving humans and was in line with the protocol approved by the Ethics Committee of the First Hospital of Lanzhou University (Gansu, China).

### Reverse transcription quantitative real-time polymerase chain reaction (RT-qPCR)

Total RNA was extracted from the cells using TRIzol® reagent (Invitrogen, Thermo Fisher Scientific, Inc.) as previously described [2]. After RNA was reverse transcribed into cDNA using PrimeScript™ RT kit (Takara, Otsu, Japan), mRNA levels were assessed with RT-qPCR using SYBR Premix EX Taq™ kit (Takara, Otsu, Japan). The primers for RT-qPCR are listed in Supplementary file 1: Table S1. The gene expression level was normalized to the endogenous reference gene β-actin. The primers for miR-1246 and U6 were purchased from Shanghai GenePharma Co., Ltd (Shanghai, China) and listed in Supplementary file 1: Table S2. The expression level of miRNAs was detected using Hairpin-it™ miRNAs qPCR Quantitation Kit (GenePharma, Shanghai, China) and normalized to the U6 endogenous control and calculated using the 2^−ΔΔCt^ method.

### miRNA and siRNA transfection

The sequences of miR-1246 mimics, miR-1246 inhibitor (in-miR-1246), mimics control (NC mimics) and inhibitor control (NC inhibitor, in-NC), siRNA NC (si-NC) and CADM1 siRNA (si-CADM1) were listed in Supplementary file 1: Table S3 and S4. They were designed and synthesized by GenePharma (Shanghai, China) and performed as previously described according to the manufacturer’s instructions [2]. After the cells were seeded at a density of 5 × 10^5^ cells/well in 6-well plates, they were transfected using Lipofectamine 2000 (Invitrogen; Thermo Fisher Scientific, USA) for 48 h. After transfection, the cells were collected and verified using RT-qPCR or Western blot, and the successfully transfected cells were used for subsequent experiments.

### Cell viability assay

The growth curve experiments were determined by (3-[4,5-dimethylthiazol-2-yl]-2,5 diphenyl tetrazolium bromide) assay (MTT assay) at 0, 24, 48, 72, 96 h after seeding. Cell viability assay was performed as previously described. In brief, the cells in the logarithmic growing phase were seeded in 96-well plates at a density of 9 × 10^3^ cells/well. After the specified time, 10 µl MTT was added into each well and the optical density at 570 nm was measured using a Powerwave X Plate reader (Bio-Tek Instruments, Inc.). The cell viability was calculated.

### 5-Ethynyl-20-deoxyuridine (EdU) incorporation assay

EdU incorporation assay was used to detect the proliferation ability of cells and executed according to the manufacturer’s protocol (BeyoClick™ EdU Cell Proliferation Kit with Alexa Fluor 488, Beyotime, Shanghai, China). Briefly, the cells were seeded in 12-well plates. After treatment, each well was incubated with 10 µM EdU at 37℃, 5% CO_2_ for 2 h. Then, the cells were collected and treated sequentially with 4% paraformaldehyde for 15 min, 0.3% Triton X-100 in PBS for 15 min, 0.2 mL Click mixture solution for 30 min and Hoechst 33,342 for 10 min at room temperature. EdU incorporation rate was expressed as the ratio of EdU positive cells (green cells) to total Hoechst 33,342 positive cells (blue cells). Images of five randomly selected areas in each group were taken with a fluorescence microscope (IX81; Olympus Corporation, Tokyo, Japan).

### Flow cytometry assay

After transfection, the cells were collected and fixed in 70% ethanol overnight and stained with Propidium Iodide (PI) in the dark for 30 min at room temperature. Finally, FACSVerse flow cytometer (BD Biosciences, San Jose, CA, USA) was used to detect them. The cell cycle data were analyzed by ModFit LT software (Veirty, USA).

Cell apoptosis was determined using the Annexin V/dead cell apoptosis kit (cat. no. V13241; Invitrogen; Thermo Fisher Scientific, Inc.) according to the protocol. The cells were stained with 5 µL Annexin V-FITC and 5 µL PI in the dark for 15 min at room temperature. Apoptosis was detected by FACSVerse flow cytometer (BD Biosciences, San Jose, CA, USA). The apoptotic rate was calculated as follows: Apoptotic rate = (early apoptotic rate + late apoptotic rate) × 100%.

### Western blot assay

Total proteins were isolated from cultured cells using ice-cold Radio Immunoprecipitation Assay (RIPA) lysis buffer (Solarbio, Beijing, China) supplemented with a protease inhibitor cocktail (Solarbio, Beijing, China). After the concentrations were determined by BCA protein assay kit (Solarbio, Beijing, China), equal amounts of protein were separated on sodium dodecyl sulfate-polyacrylamide gel electrophoresis (SDS-PAGE) gels and transferred to polyvinylidene difluoride (PVDF) membranes (EMD Millipore, Bedford, MA, USA), which were blocked with 5% non-fat milk in Tris-buffered saline with Tween-20 (TBST). Then, the membranes were immunoblotted with the primary antibodies and their corresponding horseradish peroxidase (HRP)-conjugated secondary antibodies. Specific bands were detected by enhanced chemiluminescence (ECL) kit (Millipore Corporation, Billerica, MA, USA). The primary antibodies used were as follows: rabbit anti-CADM1 (1:1000; Proteintech, Chicago, In, USA), rabbit anti-Cyclin D1 (1:1000; Wanleibio, Shenyang, China), rabbit anti-Cyclin E (1:1000; Wanleibio, Shenyang, China), rabbit anti-CDK2 (1:1000; Immunoway, Plano, TX, USA), rabbit anti-CDK4 (1:1000; Immunoway, Plano, TX, USA), rabbit anti-Cleaved PARP (c-PARP) (1:1000; Cell Signaling Technology, Danvers, MA, USA) and β-actin (1:2500; Immunoway, Plano, TX, USA).

### Dual luciferase reporter assay

Dual luciferase reporter assay was performed as previously described [2]. A fragment of CADM1 3’ UTR containing the predicted binding site for hsa-miR-1246 and flanking sequence on each side was synthesized, amplified, cloned and inserted into a pmirGLO luciferase vector in sense or anti-sense directions using SacI and Xho I at the restriction enzyme cutting sites. The primers were listed in Supplementary file1: Table S5. Each vector, along with miR-1246 mimics or mimics control, was transfected into 293T cells using Lipofectamine 2000 (Invitrogen; Thermo Fisher Scientific, USA). After 48 h of cotransfection, firefly and renilla luciferase activities of each group were measured using the Dual-Glo® Luciferase Reporter Assay System (Promega, Madison, WI, USA) according to the manufacturer’s instructions. Relative luciferase activity was normalized with renilla luciferase activity.

### Statistical analyses

SPSS (version 16.0) or GraphPad Prism software (version 6.0) were used to perform statistical analyses. All experimental data were expressed as mean ± standard deviation (SD). The differences between groups were determined by t-test or one-way analysis of variance (ANOVA) followed by Turkey’s post hoc test. Statistical significance was defined as *p* < 0.05. Asterisks (* and **) or pound signs (^#^ and ^##^) stood for *p* < 0.05 and *p* < 0.01, respectively.

## Results

### CADM1 was a direct target of miR-1246 in chemo-resistant leukemia cell lines

**Fig. 1:**
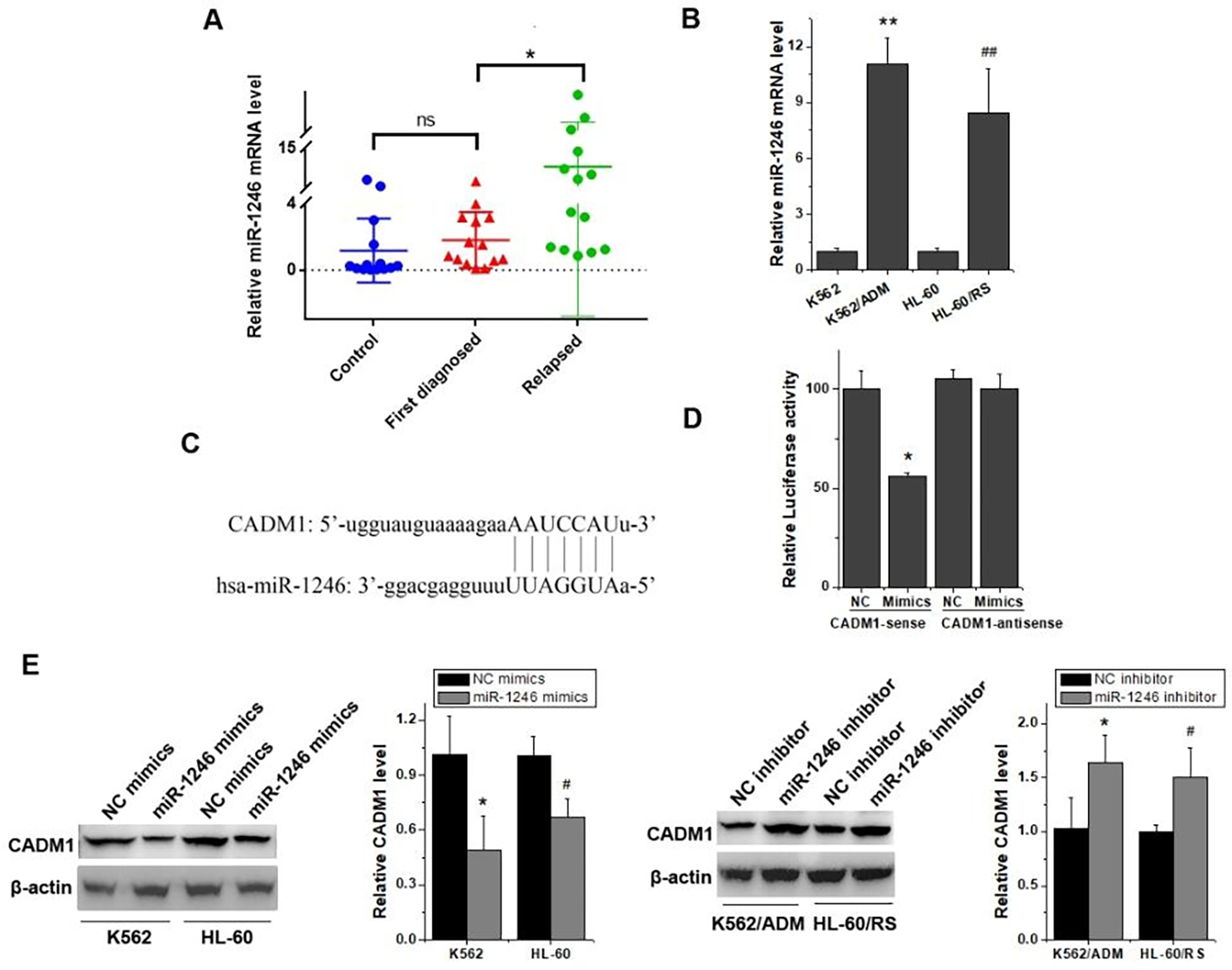
CADM1 was a direct target of miR-1246. (A) The relative miR-1246 mRNA expression level in samples from donors and patients were checked by RT-qPCR. ns, not significant; *p < 0.05 vs. the first diagnosed leukemia patients. (B) RT-qPCR was used to examine the mRNA expression level of miR-1246 in chemo-resistant leukemia cells and their parental leukemia cells. **p < 0.01 and ^##^*p* < 0.01 vs. their parental leukemia cells. (C) miRNA target prediction tools inferred that CADM1 was a potential target of miR-1246. (D) Dual luciferase reporter assay was carried out to detect the targeting relationship between miR-1246 and CADM1. *p < 0.05 vs. the NC mimics + CADM1 sense group. (E) Western blot was carried out to compare the CADM1 expression differences in chemo-resistant leukemia cells transfected with miR-1246 inhibitor or NC inhibitor and in parental sensitive leukemia cells transfected with miR-1246 mimics or NC mimics. *p < 0.05 and ^#^*p* < 0.05 vs. its NC mimics or NC inhibitor group

The results of RT-qPCR showed that the expression level of miR-1246 in first diagnosed leukemia patients was slightly higher than that of donors, but the difference was not statistically significant (Fig. 1A). What’s more surprising is that notably elevated expression level of miR-1246 was observed in relapsed blood samples than in first diagnosed leukemia blood samples (Fig. 1A). Consistent with the phenomena observed in clinical samples, the relative miR-1246 mRNA levels of chemo-resistant leukemia cells also increased remarkably than those of their parental leukemia cells (Fig. 1B). Further experiments were conducted to clarify the mechanism. Several miRNA target prediction tools, including Targetscan (http://www.targetscan.org/), miRDB (http://www.mirdb.org/), miRanda (http://www.microrna.org/), miRWalk (http://mirwalk.umm.uni-heidelberg.de/), DIANA (http://diana.imis.athena-innovation.gr/DianaTools/index.php), indicated that CADM1 was one of the direct target genes of miR-1246. Further analysis by bioinformatics tools stated clearly that a fragment at the 3’ UTR of CADM1 had a putative seed-matching site with miR-1246 (Fig. 1C). Dual luciferase reporter assay was used to confirm the prediction. As shown in Fig. 1D, luciferase activity was strikingly reduced in miR-1246 mimics + CADM1 sense group compared with its negative control or antisense control. Further results exhibited that interference with miR-1246 by its inhibitor could enhance the protein expression levels of CADM1 in K562/ADM and HL-60/RS cells, while mimics of miR-1246 could decline CADM1 protein expressions in K562 and HL-60 cells (Fig. 1E). Altogether, the above results hinted that CADM1 might be a direct target of miR-1246 in chemo-resistant leukemia cells.

### The expression level of CADM1 decreased evidently in relapsed leukemia patients and chemo-resistant leukemia cell lines

**Fig. 2:**
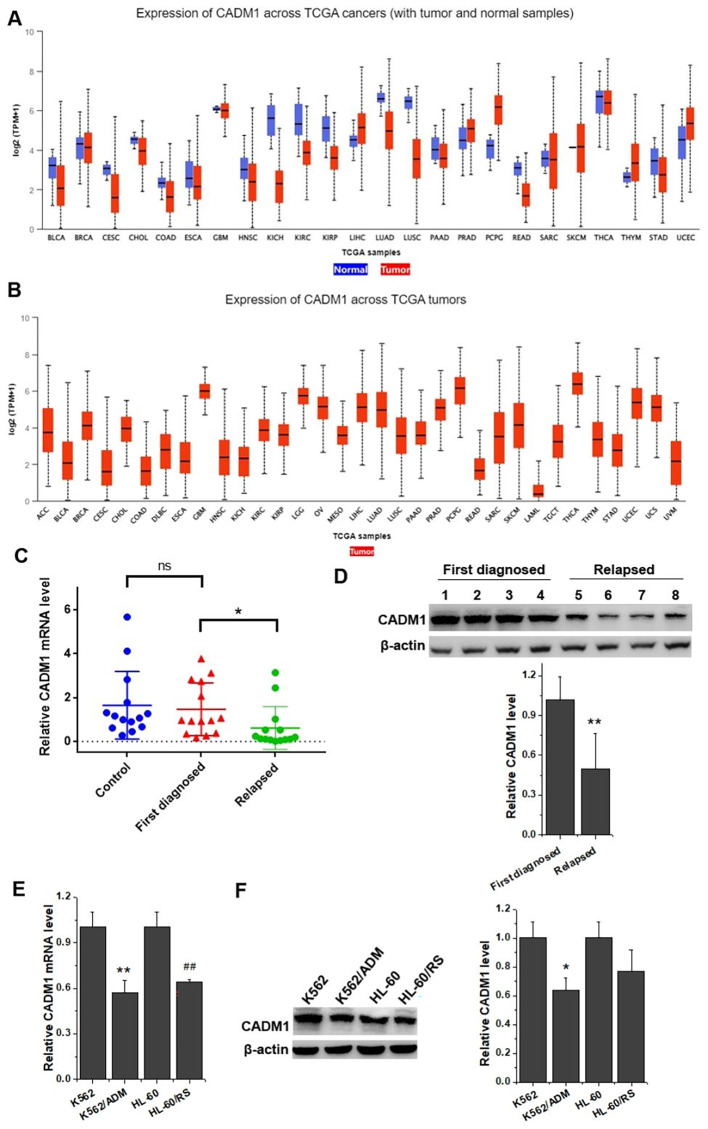
CADM1 expression was inhibited in relapsed leukemia patients and chemo-resistant leukemia cell lines. (A) The expression of CADM1 in tumor and normal samples was analyzed across TCGA cancers. BLCA, bladder urothelial carcinoma. BRCA, breast invasive carcinoma. CESC, cervical & endocervical cancer. CHOL, cholangiocarcinoma. COAD, colon adenocarcinoma. ESCA, esophageal carcinoma. GBM, glioblastoma multiforme. HNSC, head & neck squamous cell carcinoma. KICH, kidney chromophobe. KIRC, kidney renal clear cell carcinoma. KIRP, kidney renal papillary cell carcinoma. LIHC, liver hepatocellular carcinoma. LUAD, lung adenocarcinoma. LUSC, lung squamous cell carcinoma. PAAD, pancreatic adenocarcinoma. PRAD, prostate adenocarcinoma. PCPG, pheochromocytoma & paraganglioma. READ, rectum adenocarcinoma. SARC, sarcoma. SKCM, skin cutaneous melanoma. THCA, thyroid carcinoma. THYM, thymoma. STAD, stomach adenocarcinoma. UCEC, uterine corpus endometrioid carcinoma. (B) The expression of CADM1 was extracted from TCGA tumors. ACC, adrenocortical cancer. DLBC, diffuse large B-cell lymphoma. LGG, brain lower grade glioma. OV, ovarian serous cystadenocarcinoma. MESO, mesothelioma. LAML, acute myeloid leukemia. TGCT, testicular germ cell tumor. UCS, uterine carcinosarcoma. UVM, uveal melanoma. (C) The relative CADM1 mRNA expression level was detected by RT-qPCR in samples from donors and patients. ns, not significant; *p < 0.05 vs. the first diagnosed leukemia patients. (D) The protein expression level of CADM1 was examined by Western blot. **p < 0.01 vs. the first diagnosed leukemia patients. (E) RT-qPCR was carried out to test the CADM1 mRNA expression level in chemo-resistant K562/ADM and HL-60/RS cells and their parental leukemia cells. **p < 0.01 vs. K562; ^##^*p* < 0.01 vs. HL-60. (F) Western blot was used to measure the protein expression levels of CADM1 in chemo-resistant leukemia cells and their parental leukemia cells. *p < 0.05 vs. K562

As a tumor regulator, the cell adhesion molecule CADM1 is involved in cell adhesion and signal transduction, and produces a certain effect on the occurrence and development of tumors [11]. As shown by TCGA database analysis (Fig. 2A), the expression of CADM1 was obviously downregulated in several solid tumor cells. However, few studies reported CADM1 in leukemia for its complexity, and TCGA database only showed that its expression in acute myeloid leukemia (LAML) was notably lower than that in other tumors (Fig. 2B). In order to clarify the relationship among CADM1, chemotherapy resistance and leukemia, RT-qPCR and Western blot were used to determine CADM1 expression in leukemia patients and cells with or without chemo-resistance. Compared with donors, the mRNA expression level of CADM1 in first diagnosed leukemia patients was decreased. Even lower levels of CADM1 expression could be found in the blood of patients with partial or complete relapsed leukemia than that of patients with first diagnosed leukemia (Fig. 2C). Our results from Western blot displayed that the relative expression of CADM1 in relapsed patients was clearly lower than that in the first diagnosed leukemia patients (Fig. 2D and Supplementary Fig. 1). The similar phenomena could be observed in chemo-resistant leukemia cells and their parental leukemia cells (Fig. 2, E and F). The mRNA and protein level of CADM1 downregulated in chemo-resistant K562/ADM and HL-60/RS cells than in their parental sensitive leukemia cells. These results suggested that the expression level of CADM1 decreased in relapsed leukemia patients and chemotherapy-resistant leukemia cells.

### miR-1246 regulated cell cycle, proliferation, apoptosis and drug resistance by directly targeting CADM1

**Fig. 3:**
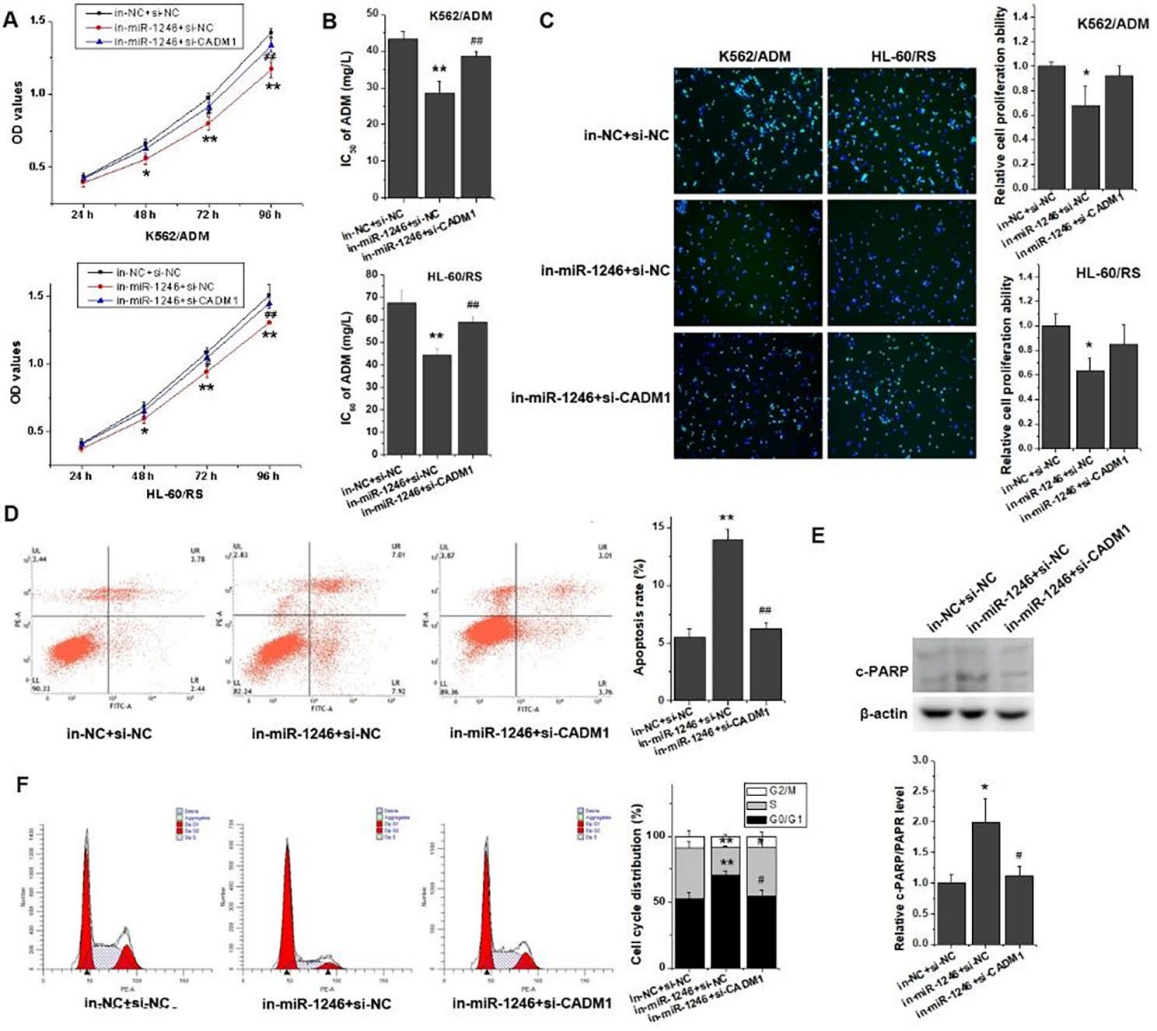
CADM1 mediated viability, drug sensitivity, proliferation, apoptosis and cell cycle distribution of chemo-resistant leukemia cells. (A) The cell viability was measured using MTT assay in chemo-resistant K562/ADM and HL-60/RS cells transfected with in-NC + si-NC, in-miR-1246 + si-NC, or in-miR-1246 + si-CADM1. (B) MTT assay was performed to detect the IC_50_ of K562/ADM and HL-60/RS cells transfected with in-NC + si-NC, in-miR-1246 + si-NC, or in-miR-1246 + si-CADM1 to ADM. (C) EdU incorporation assay was used to examine the cell proliferation ability of K562/ADM and HL-60/RS cells transfected with in-NC + si-NC, in-miR-1246 + si-NC, or in-miR-1246 + si-CADM1. (D) The cells were stained with Annexin V-FITC and PI, and the cell apoptotic rate was tested by flow cytometry. (E) The relative c-PARP expression level was detected by Western blot. (F) After the cells were fixed and stained, flow cytometry was used to check the cell cycle distribution in K562/ADM transfected with in-NC + si-NC, in-miR-1246 + si-NC, or in-miR-1246 + si-CADM1. *p < 0.05 and **p < 0.01 vs. the in-NC + si-NC group; ^#^*p* < 0.05 and ^##^*p* < 0.01 vs. the in-miR-1246 + si-NC group

Previously, we found that interference with miR-1246 had a reversal effect on the multidrug resistant ability and improved sensitivity of K562/ADM and HL-60/RS cells to ADM by inhibiting proliferation, blocking the cell cycle distribution, inducing cell apoptosis [2]. Some rescue experiments were conducted by transfecting in-NC + si-NC, in-miR-1246 + si-NC, or in-miR-1246 + si-CADM1 into chemo-resistant leukemia cells to illuminate whether CADM1 participated in the process of miR-1246 playing its biological function in chemotherapy-resistant leukemia cells. Consistent with the preliminary experimental results, inhibition of miR-1246 could restrain the cell viability, drug resistance, proliferation ability, induce cell apoptosis and block cell cycle progression at G0/G1 phase (Fig. 3, A-F). Rescue experiments revealed that interference with CADM1 could markedly heighten the cell viability, drug resistant ability to ADM and alleviate the decrease of cell activity caused by miR-1246 inhibition in chemo-resistant leukemia cells (Fig. 3, A and B). EdU incorporation assay also proved that silencing with CADM1 could relieve the impaired cell proliferation ability caused by interference with miR-1246 (Fig. 3C). Knocking down CADM1 in in-miR-1246 group could suppress the cell apoptotic rate (Fig. 3, D and E). Compared with in-miR-124 + si-NC transfected cells, cell cycle distribution analysis displayed that the proportion of in-miR-1246 + si-CADM1 transfected cells rose clearly in S phase and diminished obviously in G0/G1 phase (Fig. 3F), implying that weakened CADM1 could reverse the inhibitory effect of miR-1246 suppression on cell cycle process and restore cell cycle progression. These results suggested that down-regulated CADM1 mediated high proliferation, low apoptosis and strong drug resistance of chemo-resistant leukemia cells. Thus, CADM1 played an indispensable mediating role in the biological function of miR-1246 on leukemia.

### Down-regulated CADM1 along with up-regulated miR-1246 mediated cell cycle progression via CDKs/Cyclins complexes

**Fig. 4:**
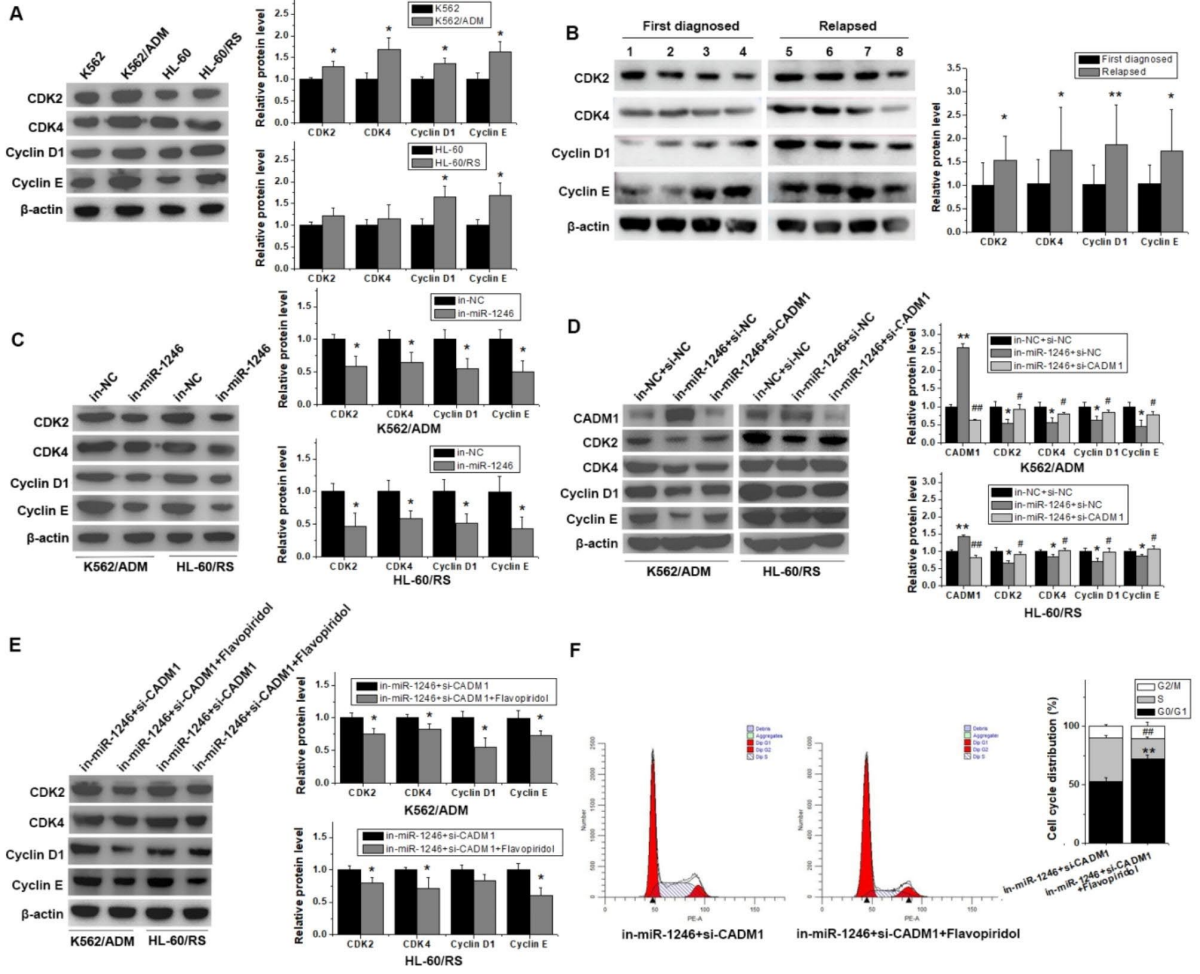
miR-1246/CADM1 axis regulated cell cycle progression by tuning up CDKs. (A) and (B) The protein levels of CDK2, CDK4, Cyclin E and Cyclin D1 were detected in chemo-resistant leukemia cells, their parental sensitive leukemia cells, and clinical samples. *p < 0.05 and **p < 0.01 vs. their parental leukemia cells or the first diagnosed leukemia patients. (C) Comparing to NC-inhibitor transfected chemo-resistant leukemia cells, the protein levels of CDK2, CDK4, Cyclin E and Cyclin D1 in miR-1246 inhibitor transfected chemo-resistant leukemia cells were analyzed. *p < 0.05 vs. the in-NC group. (D) The protein immunoblotting was applied to test the protein expression of CADM1, CDK2, CDK4, Cyclin E, Cyclin D1 in chemo-resistant leukemia cells transfected with in-NC + si-NC, in-miR-1246 + si-NC, or in-miR-1246 + si-CADM1. *p < 0.05 and **p < 0.01 vs. the in-NC + si-NC group; ^#^*p* < 0.05 and ^##^*p* < 0.01 vs. the in-miR-1246 + si-NC group. (E) Western blot assay was used to detect the protein levels of cell cycle related proteins CDK2, CKD4, Cyclin E, Cyclin D1. *p < 0.05 vs. the in-miR-1246 + si-CADM1 group. (F) Flow cytometer combined PI was used to examine the cell cycle distribution of K562/ADM cells transfected with in-miR-1246 + si-CADM1 with or without flavopiridol. **p < 0.01 and ^##^*p* < 0.01 vs. the in-miR-1246 + si-CADM1 group

To figure out how miR-1246 and CADM1 participated in the regulation of cell cycle distribution in chemo-resistant leukemia cells, the cell cycle related genes, including CDK2, CDK4, Cyclin D1 and Cyclin E, were focused on in the presence or absence of miR-1246 inhibition or interference with CADM1 in chemo-resistant or -sensitive leukemia cells. Compared with parental sensitive leukemia cells, the protein expression levels of CDK2, CDK4, Cyclin D1 and Cyclin E in chemo-resistant leukemia cells were relatively elevated (Fig. 4A). Similar results could also be displayed in the patient samples (Fig. 4B and Supplementary Fig. 1). The relative protein levels of CDKs in relapsed leukemia patients were higher than those in the first diagnosed leukemia patients. miR-1246 suppression could abate their protein expression level in chemo-resistant K562/ADM and HL-60/RS leukemia cells (Fig. 4C). When the activity of CADM1 was restrained in in-miR-1246 group (in-miR-1246 + si-CADM1 group), the protein expression levels of CADM1 were strikingly attenuated, while CDK2, CDK4, Cyclin E and Cyclin D1 were distinctly promoted (Fig. 4D). These results hinted that miR-1246 could weaken the expression of CADM1, which might affect cell cycle distribution of chemo-resistant leukemia cells via CDKs/Cyclins.

To confirm whether CDKs mediated miR-1246/CADM1 axis in cell cycle regulation in chemo-resistant leukemia cells, flavopiridol was used to restrict the activity of CDK2 and CDK4. Results demonstrated that the expression levels of Cyclin E and Cyclin D1 slid apparently in in-miR-1246 + si-CADM1 + flavopiridol groups than in in-miR-1246 + si-CADM1 groups (Fig. 4E). Cell cytometry analysis proved that the cell cycle progression in in-miR-1246 + si-CADM1 + flavopiridol groups were also arrested visibly (Fig. 4F). As the activity of CDK2 and CDK4 were lessened, the proportion of cells in G0/G1 phase enhanced considerably, while the proportion of cells in S phase decreased simultaneously. These results implied that miR-1246 dominated cell cycle progression in chemo-resistant leukemia cells by controlling CADM1/CDK/Cyclin axis.

## Discussion

Leukemia, the most common cancer in children, takes its rise from clonal expansion of abnormal progenitor cells, and acts out increased proliferation and failed differentiation. It is usually gauged by uncontrollability of blood cells in the bone marrow and subsequent peripheral blood [12, 13]. The interruption of normal cell cycle execution plays an important part in the development of leukemia. Generally, the cell cycle is a strictly regulated process, and the cell cycle checkpoints guarantee that the progress of the cell cycle from one phase to another is precisely adjusted and occurs with high fidelity. Dysregulation of the cell cycle checkpoints in G1/S, S, G2, and M-phase could lead to abnormal cell proliferation and carcinogenesis [14]. The main regulation of the cell cycle is achieved through a series of cyclins and cyclin dependent kinases (CDKs). Each CDK demands its corresponding cyclin counterpart for stability, activity, and downstream phosphorylation. There are at least 20 CDKs and 29 cyclins [15]. A variety of cyclins, such as Cyclin D and Cyclin E, are closely related to cancer. Researches showed that Cyclin E, which bound to CDK2 and took part in S-phase initiation, was abundant in 27% of AML patients, especially in patients with M5 subtype. Its overexpression was also associated with reduced remission and overall survival. With the high abundance of Cyclin D1 and CDK4/6, cells could escape from critical cell checkpoints, then grow out of control and develop into malignant tumor cells [12]. Moreover, upregulated Cyclin E not only accelerated the proliferation and division of tumor cells, but also induced the expression of related products, leading to chemotherapy resistance. Over the years, studies had pointed out that Cyclin E went hand in hand with the chemo-resistance of tumor cells, and its expression in tumor cells was closely related to prognosis [16]. In addition, Decker et al. reported that high expression of Cyclin E had a correlation with the subtype of invasive breast cancer, poor prognosis and trastuzumab resistance [17]. Chu et al. indicated that abundant Cyclin E could mediate the drug resistance of tumor cells to various therapeutic drugs [18]. Similarly, the amplification of Cyclin D1 in multiple myeloma was related to the expression of multidrug resistance. Sewify et al. demonstrated that Cyclin D1 positive patients had remarkably lower progression free survival and overall survival, and higher MDR1 levels comparing with Cyclin D1 negative patients [19]. Our present study proved that the expression levels of CDK2, CDK4, Cyclin D1 and Cyclin E were more abundant in the chemo-resistant leukemia cells than in their parental cells (Fig. 4A). Similar phenomena could be observed in the patient samples. The relative protein levels of CDKs in relapsed leukemia patients were higher than those in the first diagnosed leukemia patients (Fig. 4B and Supplementary Fig. 1). Our above results suggest that higher expression levels of Cyclin D1 and Cyclin E were closely related to chemotherapy tolerance and might be one of the reasons for drastic drug resistance in leukemia cells.

CADM1, a member of immunoglobulin superfamily and its characterization as a cell adhesion molecule, is also named as Necl-2 and SynCAM1 and plays significant roles in cancer, neurology and immunology [20, 21]. It is originally identified as a tumor suppressor gene TSLC1 in human non-small cell carcinoma and a growing body of researches show that CADM1 has a regulatory effect on the progression of tumors [22]. It was reported to be notably downregulated in multiple human cancer tissues and cell lines, including esophageal cancer, cutaneous melanoma, hepatocellular carcinoma, ovarian carcinoma, breast cancer, pancreatic ductal adenocarcinoma, lung cancer, laryngeal squamous cell carcinoma, colorectal cancer, prostate cancer, neuroblastoma and nasopharyngeal carcinoma [11]. However, unlike the above situation, CADM1 was ample in some cancer types. Researches revealed that CADM1 was upregulated in Kaposi’s sarcoma, HTLV-I-transformed cell and adult T-cell leukemia, small cell lung cancer, Merkel cell carcinoma, mycosis fungoides, Sezary syndrome [23–25]. Nevertheless, evidences had stated clearly that the loss of CADM1 expression is interrelated with histological grades and cancer prognosis, the lower the CADM1, the stronger the tumor aggressiveness and the worse the prognosis [11]. Our results exhibited that the expression level of CADM1 was lower in blood of patients with relapsed leukemia than in their first diagnosed blood samples (Fig. 2, C and D and Supplementary Fig. 1). Consistent with which, CADM1 was also downregulated in chemo-resistant leukemia cells than in their parental cells (Fig. 2, E and F). In addition, Zhang W et al. pointed out that ectopic expression of CADM1 could inhibit cell growth and negatively regulate the G1/S transition in hepatocellular carcinoma [26]. Our study discovered that CADM1 was involved in the cell cycle regulation and impaired the effect of miR-1246 on promoting cell cycle progression in chemo-resistant leukemia cells (Fig. 3F). Further study elucidated that CADM1 participated in the cell cycle regulation by affecting the expression of Cyclins and CDKs (Fig. 4, D-F). Chen et al. demonstrated that overexpression of CADM1 protein arrested cells in the G1/G0 phases of the cell cycle and decreased the expression levels of Cyclin D1, Cyclin E1 and CDK2 proteins, while CAMD1 knockdown had an opposite effect on the cell cycle distribution and the expression level of these proteins in bladder cancer [27]. Li et al. proved that reduction of CADM1 expression could increase the expression of Cyclin D1, Cyclin E and CDK6 [28]. These results were consistent with our findings, indicating that CADM1 could regulate the cell cycle progress by Cyclins and CDKs.

As a class of endogenous small non-coding single-stranded RNA molecules, miRNAs could specifically unite their target genes and develop a crucial role in many physiological and pathophysiological processes in numerous cell types. Studies have identified that specific miRNAs could regulate the cell cycle, and the loss or gain of miRNA-mediated cell cycle control could contribute to malignancy. Emerging evidences suggested that altered expression levels of miRNAs could facilitate cancer due to imbalance of cell cycle regulation [29]. miRNAs could modulate classic cell cycle control pathways by targeting the cell cycle related proteins such as CDKs, Cyclins [30]. Expression of Cyclin D1 had been demonstrated to be adjusted by several miRNAs such as let-7b and miR-19a [31, 32]. Yang et al. proved that miR-20b-5p could act as a tumor-suppressor miRNA in the pathogenesis of colon cancer via negatively regulating the Cyclin D1/CDK4/FOXM1 axis [33]. Ding et al. showed that miR-29c influenced the activity of Cyclin E-CDK2 complexes by affecting the expression of Cyclin E [34]. In lymphoma cells, miR-29 could target 3’ UTR of CDK6 and modulate the cell cycle G1/S transition and cell proliferation [35]. Our previous results had explicated that miR-1246 was preferentially overexpressed in chemo-resistant leukemia cells, and lack of miR-1246 would repress the advancement of cell cycle by halting cells at the G0/G1 stage. Further experiments in this study interpreted that a noticeable growth in the apoptotic death in in-miR-1246 groups and an enhanced proportion of cells in the G0/G1 phase of the cell cycle with a concurrent reduction of cells in the S phase (Fig. 3, D-F). Comparing with their parental sensitive leukemia cells, the protein expression levels of CDK2, CDK4, Cyclin E and Cyclin D1 relatively elevated in chemo-resistant leukemia cells (Fig. 4A). Inhibition of miR-1246 could lessen their expression in chemo-resistant leukemia cells (Fig. 4C). These results implied that abundant miR-1246 in chemo-resistant leukemia cells might directly or indirectly activate genes related to cell cycle regulation, and finally influence the chemotherapy resistance in leukemia cells.

Several miRNA target prediction tools indicated that CADM1 was one of the targets of miR-1246 (Fig. 1C), dual luciferase reporter assay manifested that it was a direct target of miR-1246 (Fig. 1D). Few literature reported the relationship between miR-1246 and CADM1. Sun et al. proved that miR-1246 was highly expressed in metastatic hepatocellular carcinoma cells and inhibition of miR-1246 effectively reduced migration and invasion by up-regulation of CADM1 [36]. Our results revealed that miR-1246 could regulate cell cycle progression through CADM1 (Fig. 3F). Further studies elucidated that miR-1246 could also accelerate cell cycle progression via CADM1/CDKs/Cyclins axis (Fig. 4, D-F). Collectively, our data confirmed that miR-1246 could influence the expression levels of CDKs/Cyclins complexes via targeting CADM1 and indirectly adjusted cell cycle. Sun et al. declared that high expression of miR-1246 combined with low expression of CADM1 might serve as a risk factor for stage1 liver cancer patients [36]. Many studies have shown that exosomal miRNAs, including miR-1246, could act as potential diagnostic biomarkers of cancer. Ogata-Kawata et al. suggested that the serum exosomal levels of seven miRNAs (let-7a, miR-1229, miR-1246, miR-150, miR-21, miR-2224, and miR-23a) were significantly higher and were promising biomarkers for non-invasive diagnosis of colon cancer [37]. Hannafon et al. demonstrated that miR-21 and miR-1246 were selectively enriched in human breast cancer exosomes, significantly elevated in plasma from patients with breast cancer, and could serve as an important companion diagnostic tool for breast cancer [38]. Further experimental researches still need to be performed to verify the feasibility of miR-1246 and CADM1 in chemo-resistant or relapsed leukemia treatments.

## Conclusions

Taken together, CADM1, negatively correlated with miR-1246, was deficient in drug-resistant leukemia cells and relapsed leukemia patients. Interference with CADM1 could significantly attenuate the cell cycle arrest effect of miR-1246 suppression on chemo-resistant leukemia cells via influencing CDKs/Cyclins axis. Therefore, higher expression of miR-1246 combined with lower expression of CADM1 might serve as a potential therapeutic target for patients with clinically chemo-resistant and relapsed leukemia.

### Electronic supplementary material

Below is the link to the electronic supplementary material.

Supplementary Material 1

Supplementary Material 2

## Data Availability

The datasets used and/or analysed during the current study are available from the corresponding author on reasonable request.
