# Supplementary material for: CADM1 impairs the effect of miR-1246 on promoting cell cycle progression in chemo-resistant leukemia cells
Source: BMC Cancer. 2023 Oct 9;23:955. doi: 10.1186/s12885-023-11458-1 (PMC10561441; doi:10.1186/s12885-023-11458-1)
Supplement: Supplementary file 1 — Supplementary Material 1 [file 12885_2023_11458_MOESM1_ESM.pdf]

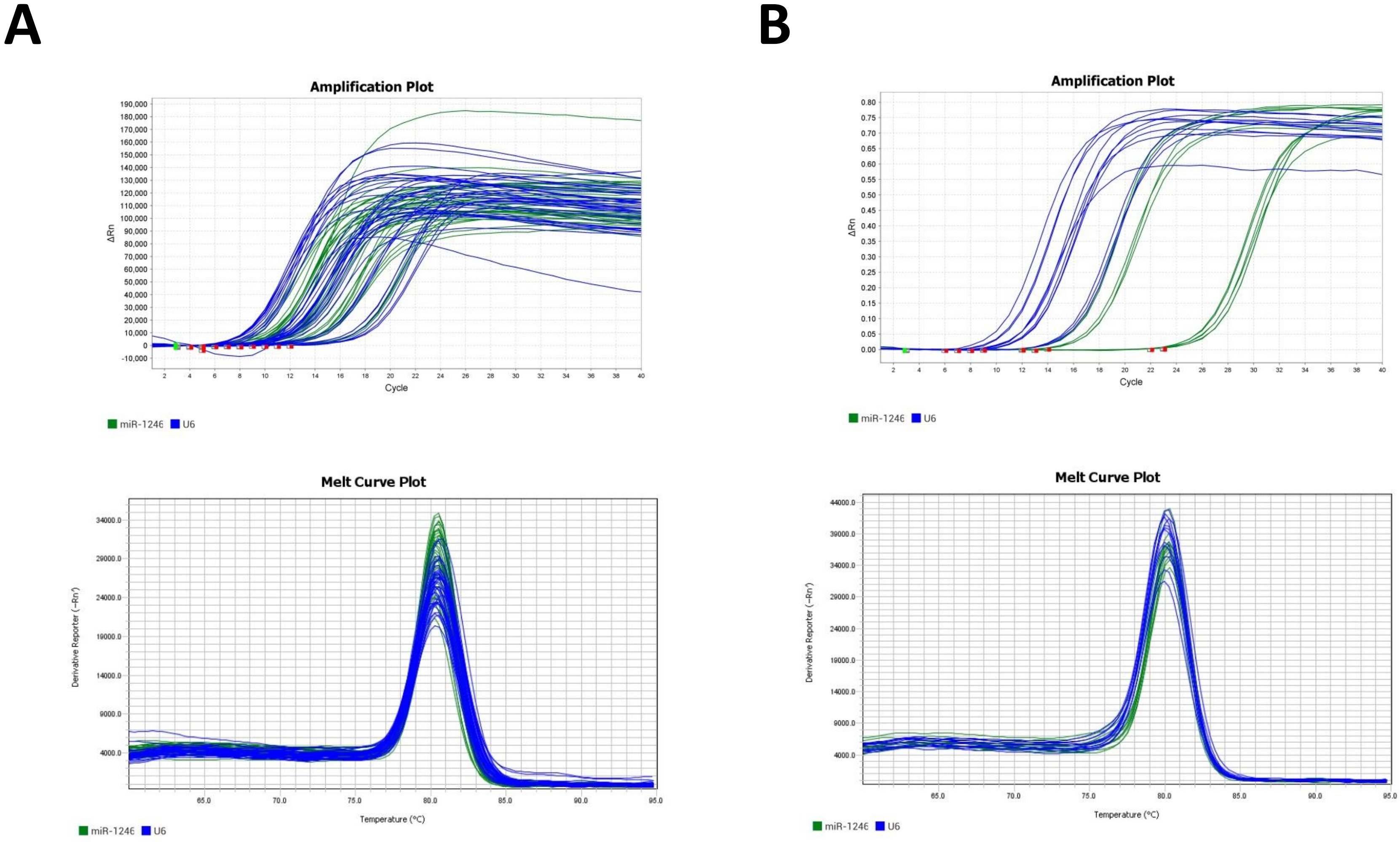
A
B
Amplification Plot
ΔRn
Cycle
miR-1246
U6
Melt Curve Plot
Derivative Reporter (−Rn')
Temperature (°C)

Figure 1

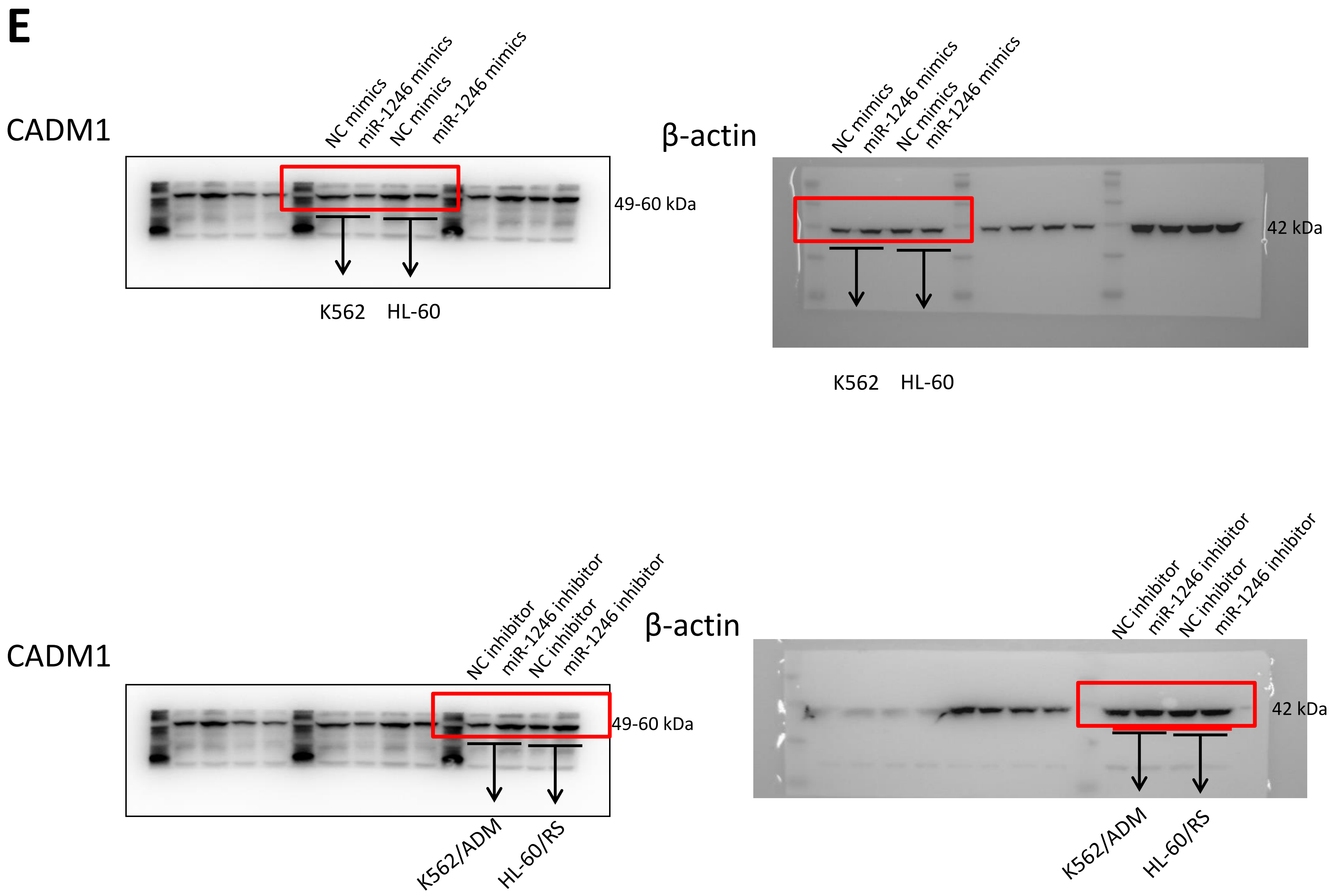
E
CADM1
NC mimics
miR-1246 mimics
NC mimics
miR-1246 mimics
49-60 kDa
K562
HL-60
β-actin
NC mimics
miR-1246 mimics
NC mimics
miR-1246 mimics
42 kDa
K562
HL-60
CADM1
NC inhibitor
miR-1246 inhibitor
NC inhibitor
miR-1246 inhibitor
49-60 kDa
K562/ADM
HL-60/RS
β-actin
NC inhibitor
miR-1246 inhibitor
NC inhibitor
miR-1246 inhibitor
42 kDa
K562/ADM
HL-60/RS

Figure 1

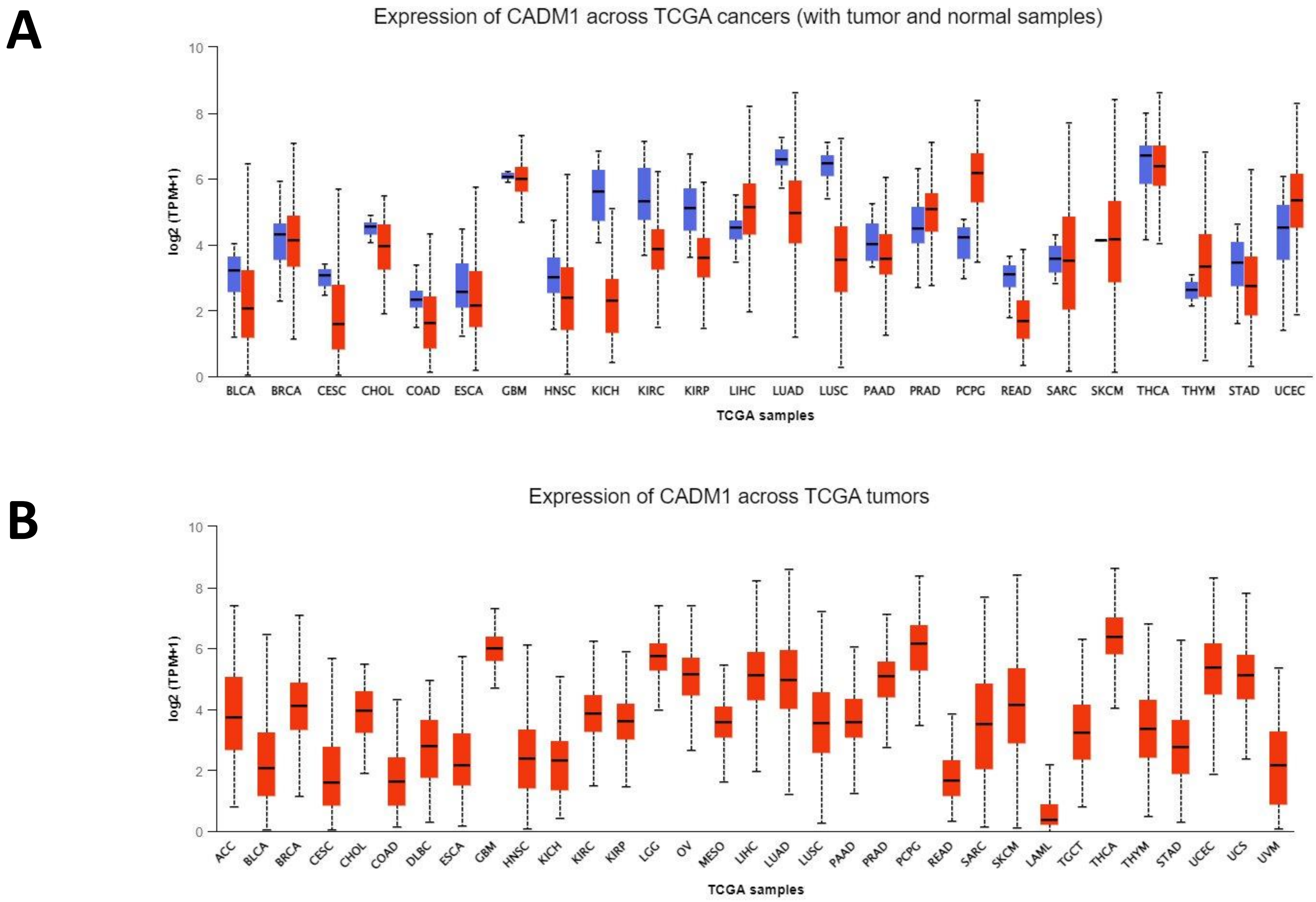
A
Expression of CADM1 across TCGA cancers (with tumor and normal samples)
log2 (TPM+1)
10
8
6
4
2
0
BLCA BRCA CESC CHOL COAD ESCA GBM HNSC KICH KIRC KIRP LIHC LUAD LUSC PAAD PRAD PCPG READ SARC SKCM THCA THYM STAD UCEC
TCGA samples
B
Expression of CADM1 across TCGA tumors
log2 (TPM+1)
10
8
6
4
2
0
ACC BLCA BRCA CESC CHOL COAD DLBC ESCA GBM HNSC KICH KIRC KIRP LGG OV MESO LIHC LUAD LUSC PAAD PRAD PCPG READ SARC SKCM LAML TGCT THCA THYM STAD UCEC UCS UVM
TCGA samples

Figure 2

C

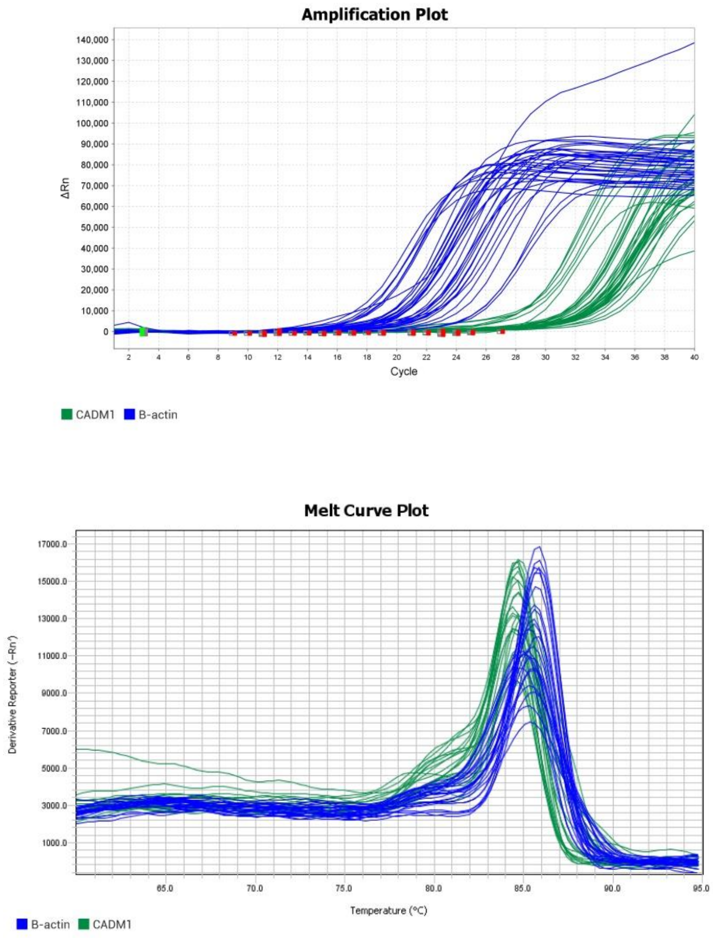
Amplification Plot
ΔRn
Cycle
CADM1
B-actin
Melt Curve Plot
Derivative Reporter (−Rn')
Temperature (°C)
B-actin
CADM1

E

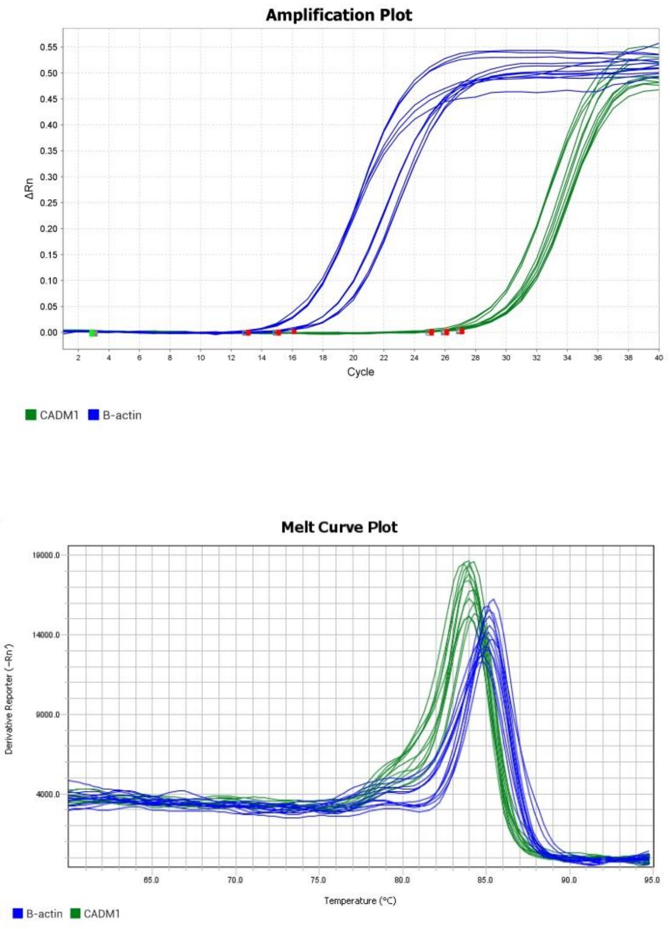
Amplification Plot
ΔRn
Cycle
CADM1
B-actin
Melt Curve Plot
Derivative Reporter (−Rn')
Temperature (°C)
B-actin
CADM1

Figure 2

D

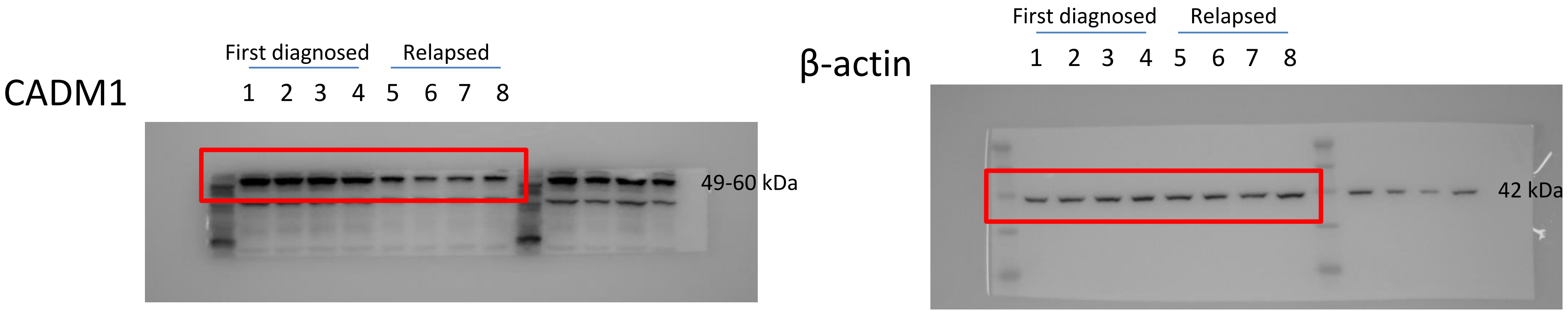
CADM1
First diagnosed
Relapsed
1 2 3 4 5 6 7 8
49-60 kDa
β-actin
First diagnosed
Relapsed
1 2 3 4 5 6 7 8
42 kDa

F

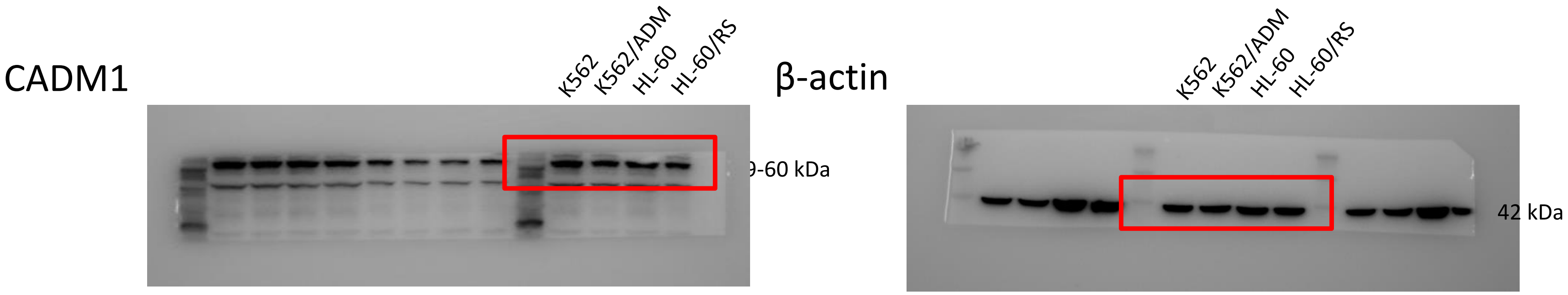
CADM1
K562
K562/ADM
HL-60
HL-60/RS
9-60 kDa
β-actin
K562
K562/ADM
HL-60
HL-60/RS
42 kDa

Figure 2

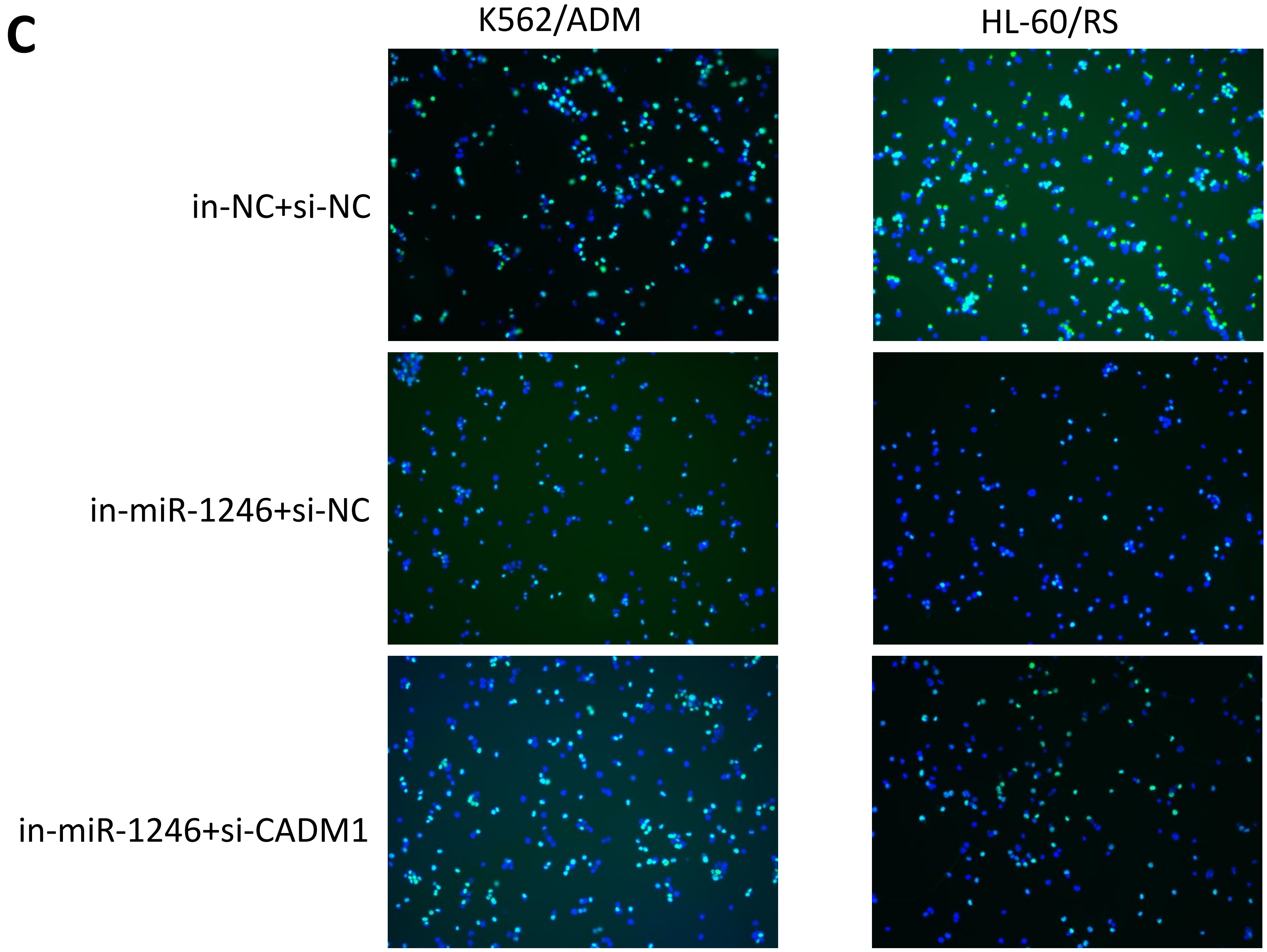
C
K562/ADM
HL-60/RS
in-NC+si-NC
in-miR-1246+si-NC
in-miR-1246+si-CADM1

Figure 3

E

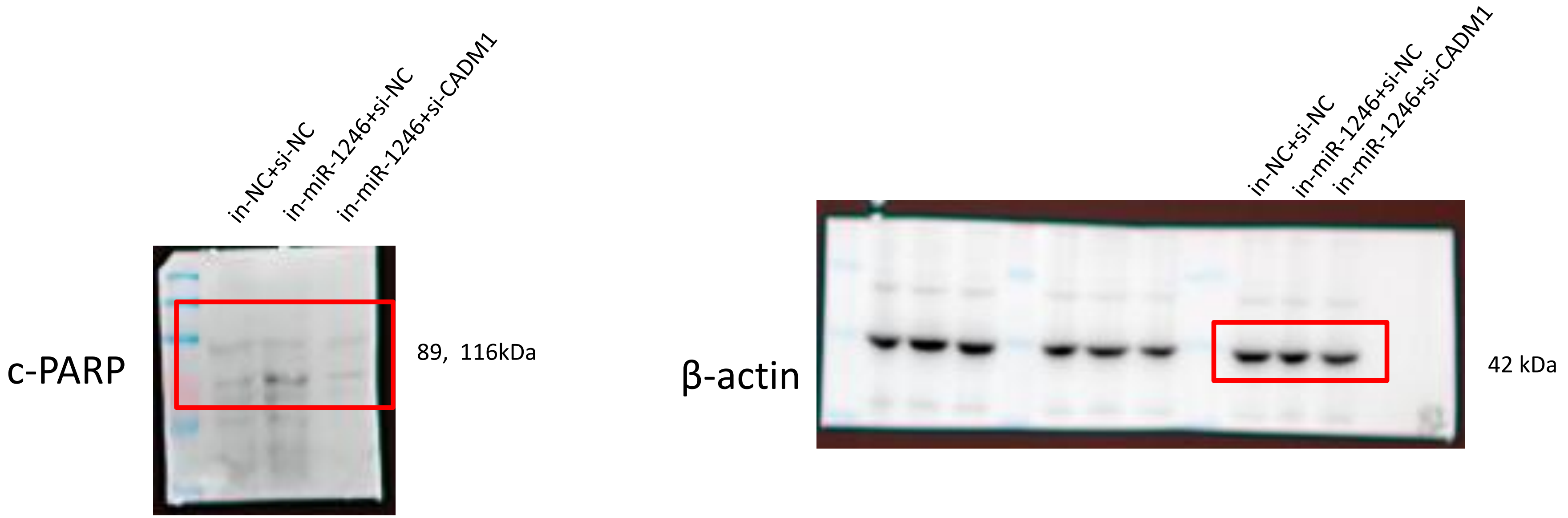
in-NC+si-NC
in-miR-1246+si-NC
in-miR-1246+si-CADM1
c-PARP
89, 116kDa
in-NC+si-NC
in-miR-1246+si-NC
in-miR-1246+si-CADM1
β-actin
42 kDa

Figure 3

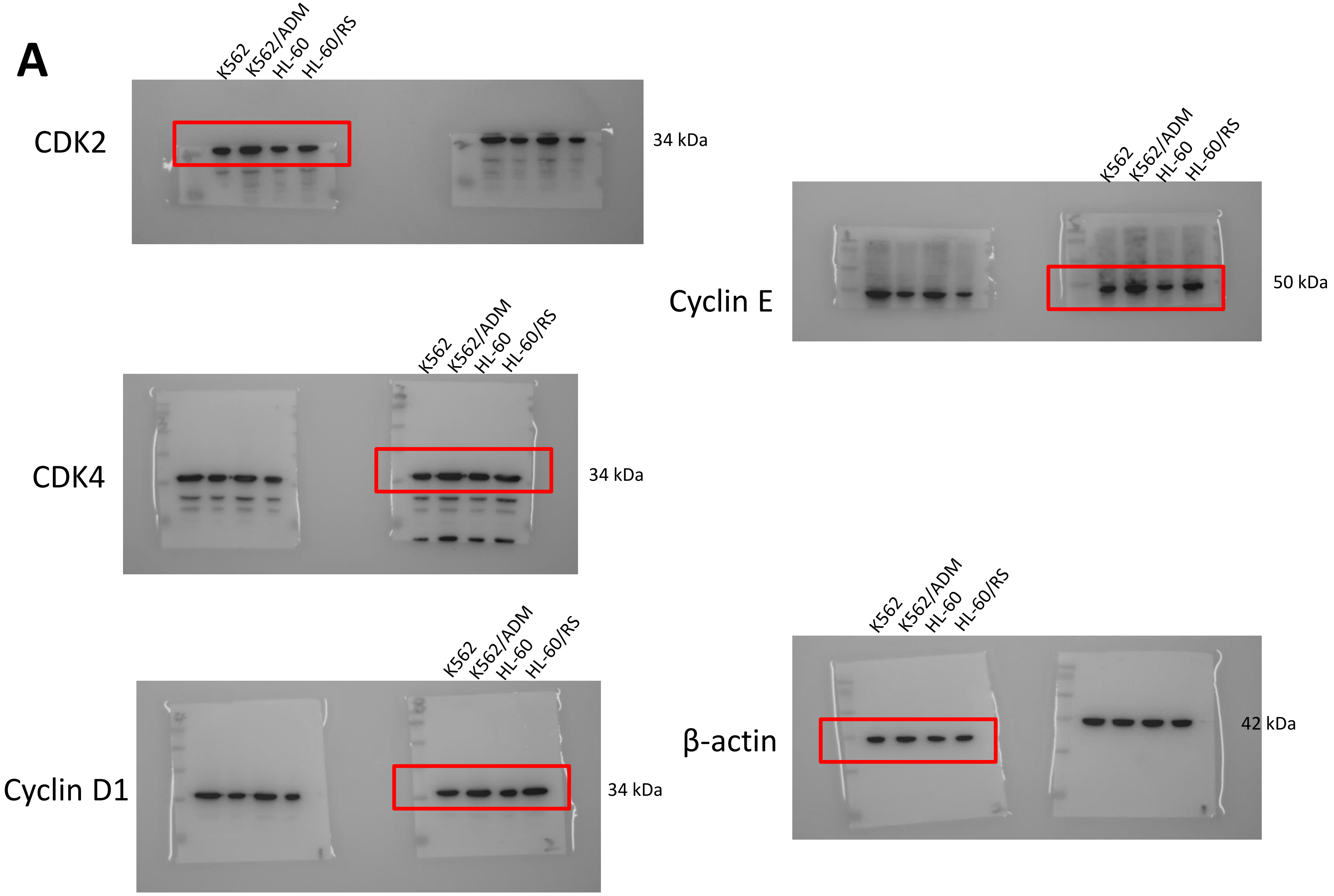
A
K562
K562/ADM
HL-60
HL-60/RS
CDK2
34 kDa
Cyclin E
50 kDa
CDK4
34 kDa
Cyclin D1
34 kDa
β-actin
42 kDa

Figure 4

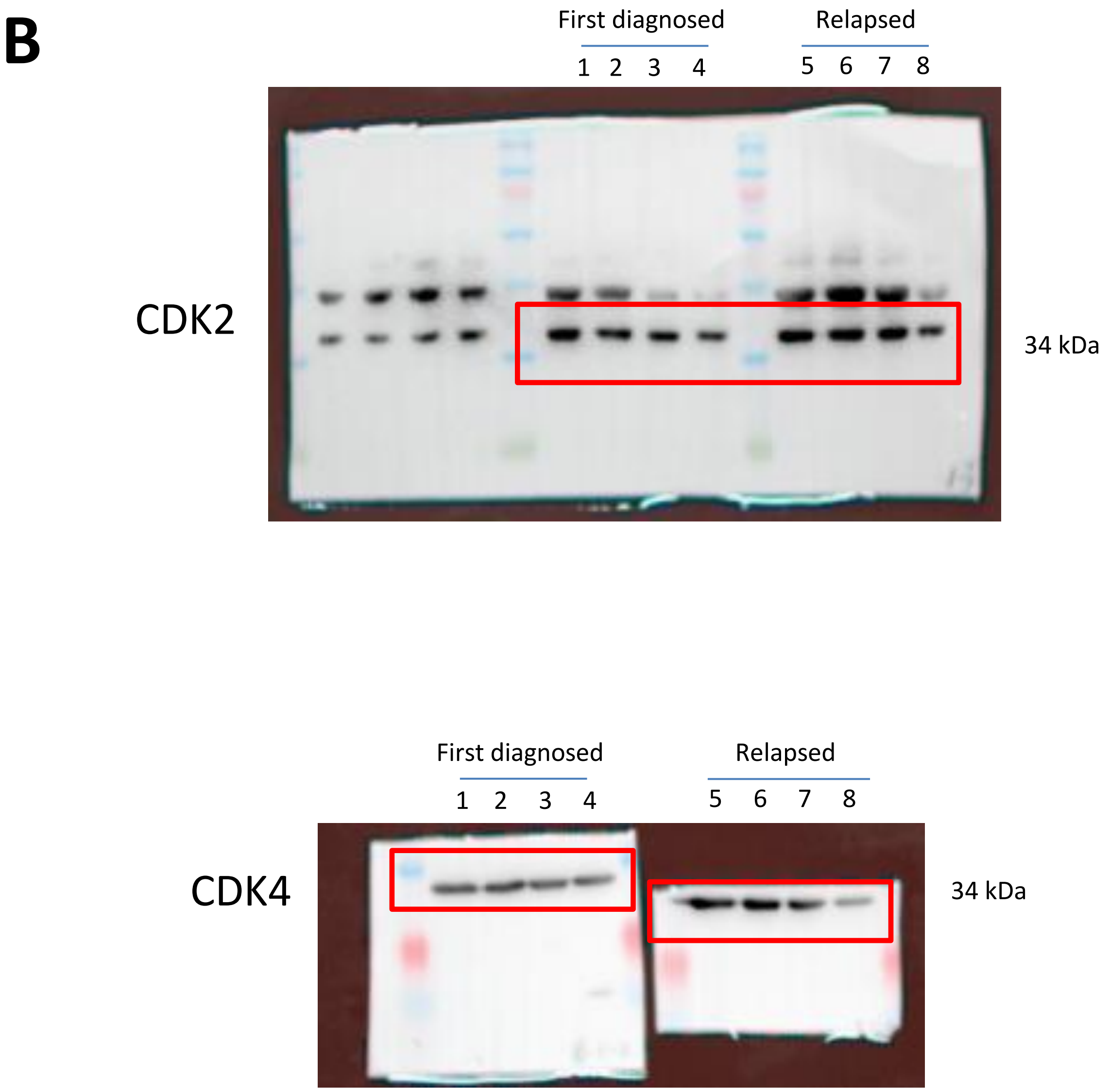
B
First diagnosed
Relapsed
1 2 3 4
5 6 7 8
CDK2
34 kDa
First diagnosed
Relapsed
1 2 3 4
5 6 7 8
CDK4
34 kDa

Figure 4

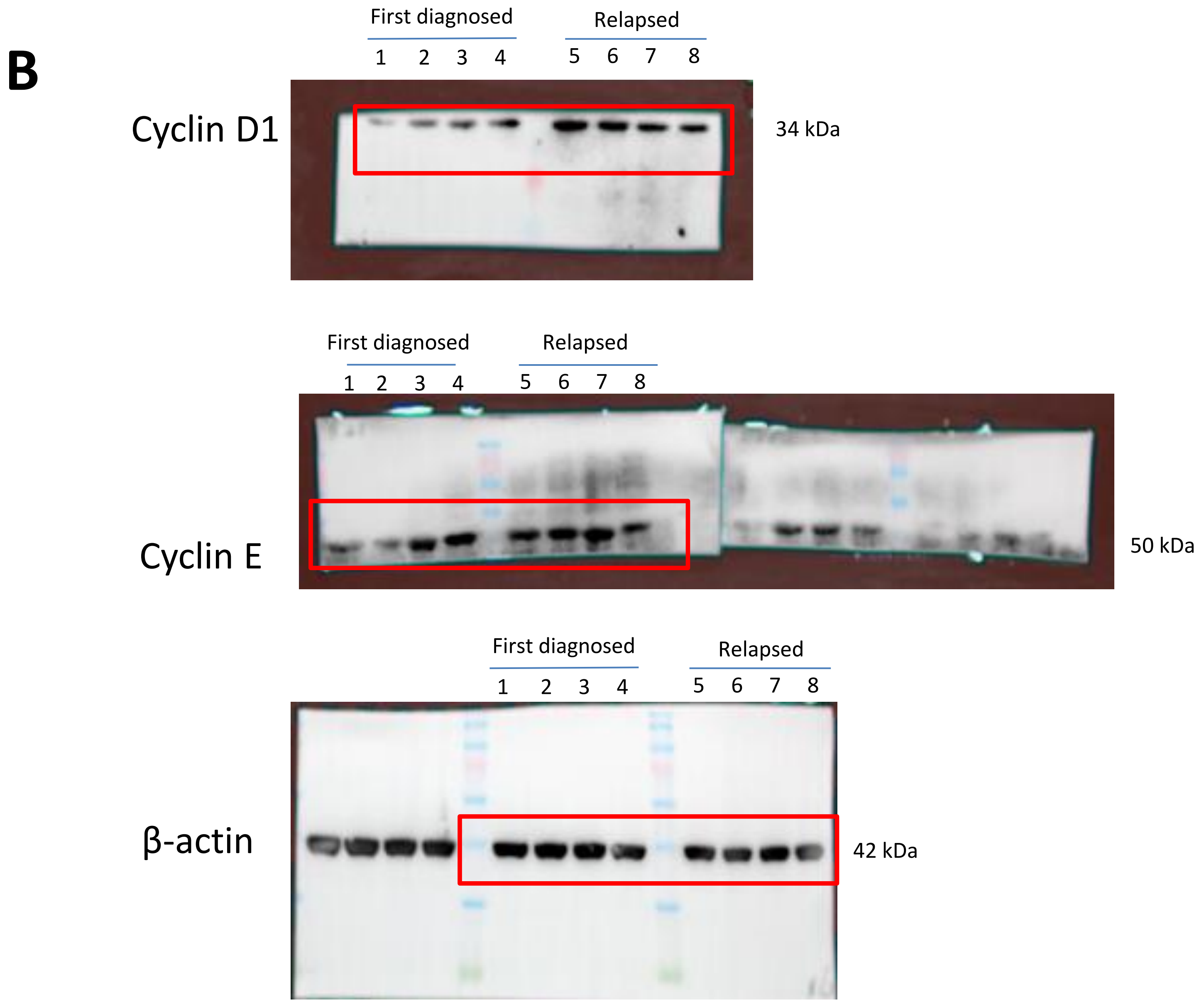
B
First diagnosed
Relapsed
1 2 3 4
5 6 7 8
Cyclin D1
34 kDa
First diagnosed
Relapsed
1 2 3 4
5 6 7 8
Cyclin E
50 kDa
First diagnosed
Relapsed
1 2 3 4
5 6 7 8
β-actin
42 kDa

Figure 4

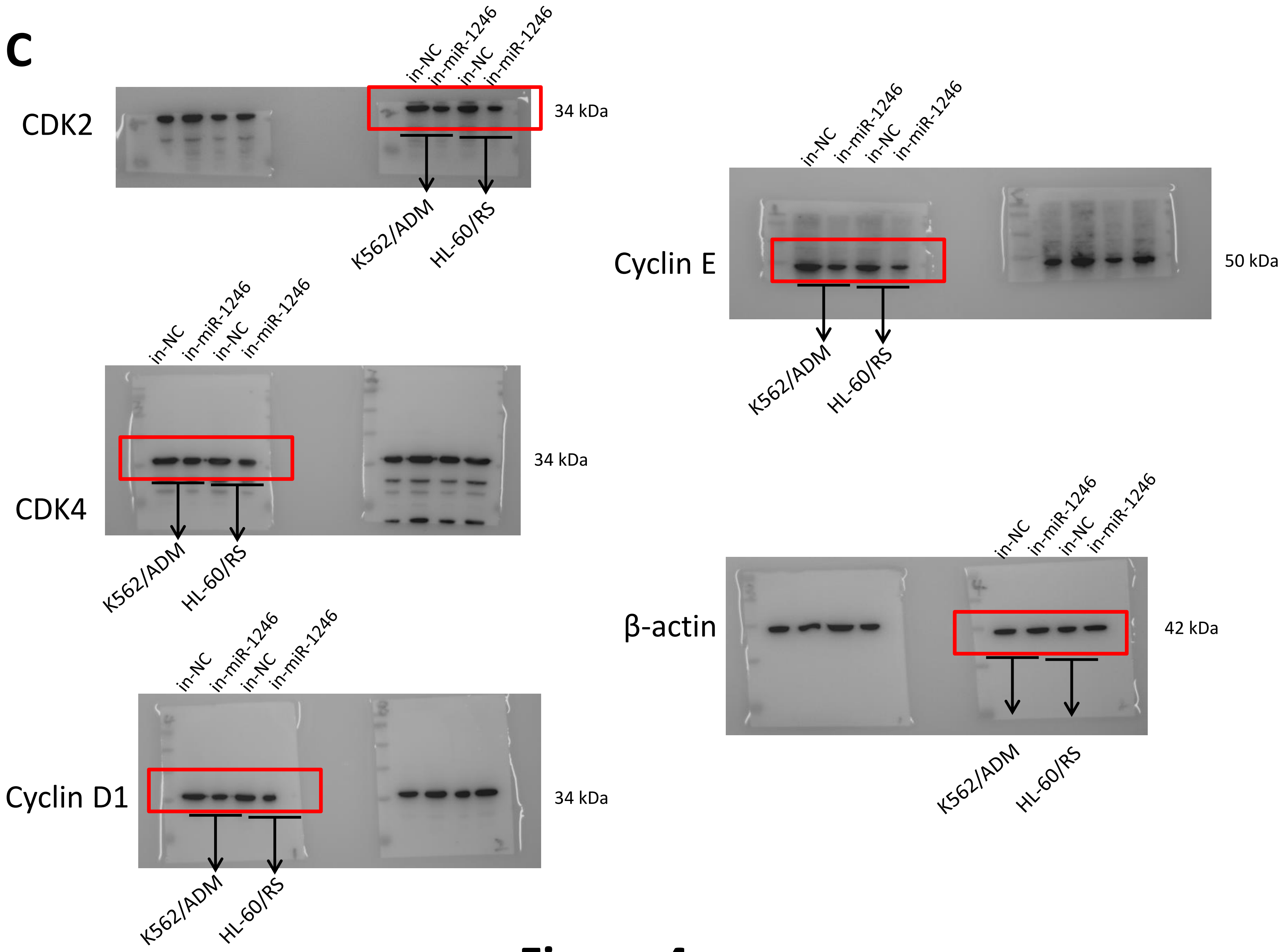
C
CDK2
in-NC
in-miR-1246
in-NC
in-miR-1246
34 kDa
K562/ADM
HL-60/RS
Cyclin E
50 kDa
CDK4
34 kDa
Cyclin D1
34 kDa
β-actin
42 kDa

Figure 4

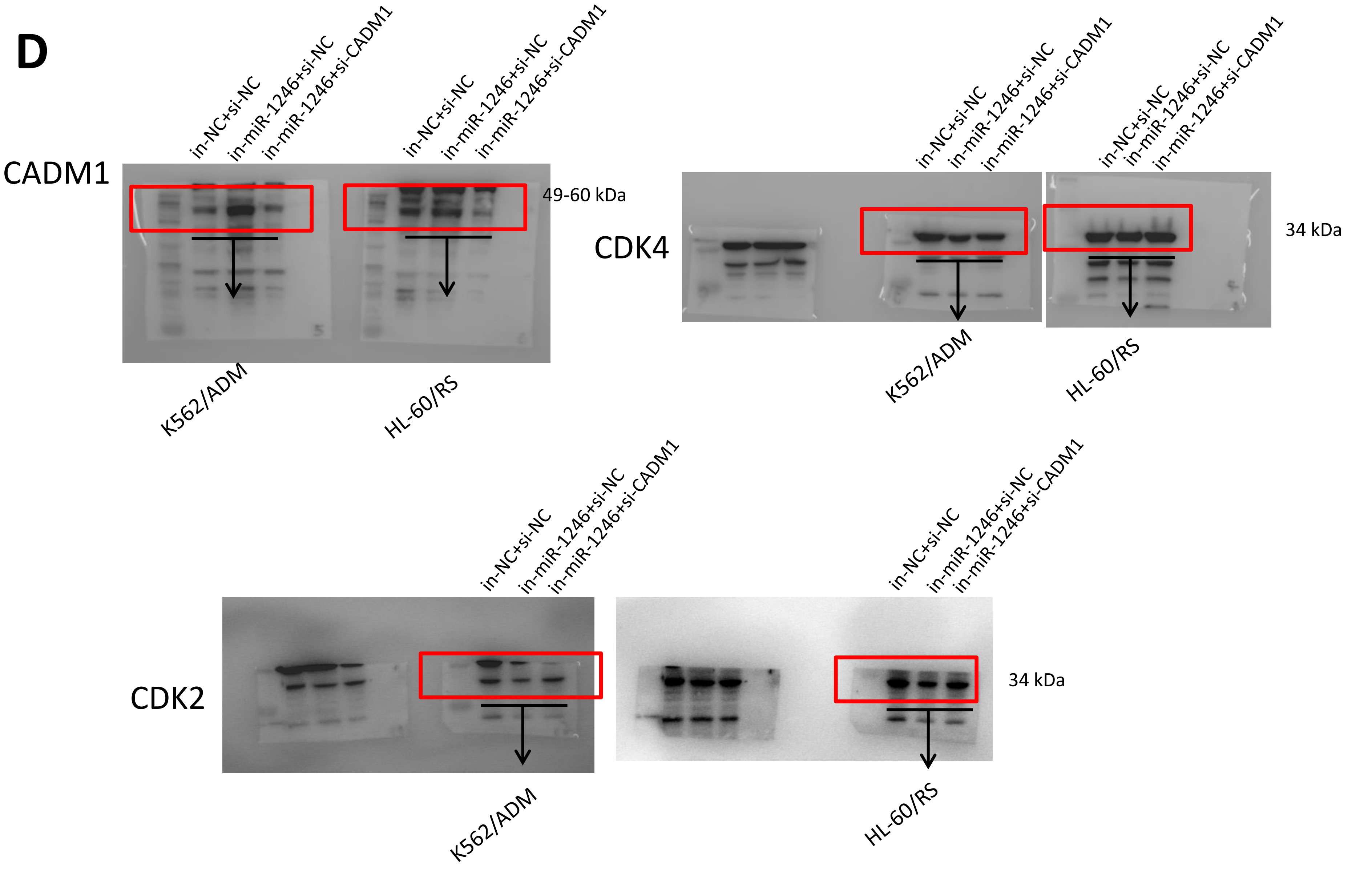
D
CADM1
in-NC+si-NC
in-miR-1246+si-NC
in-miR-1246+si-CADM1
49-60 kDa
K562/ADM
HL-60/RS
CDK4
34 kDa
CDK2
34 kDa

Figure 4

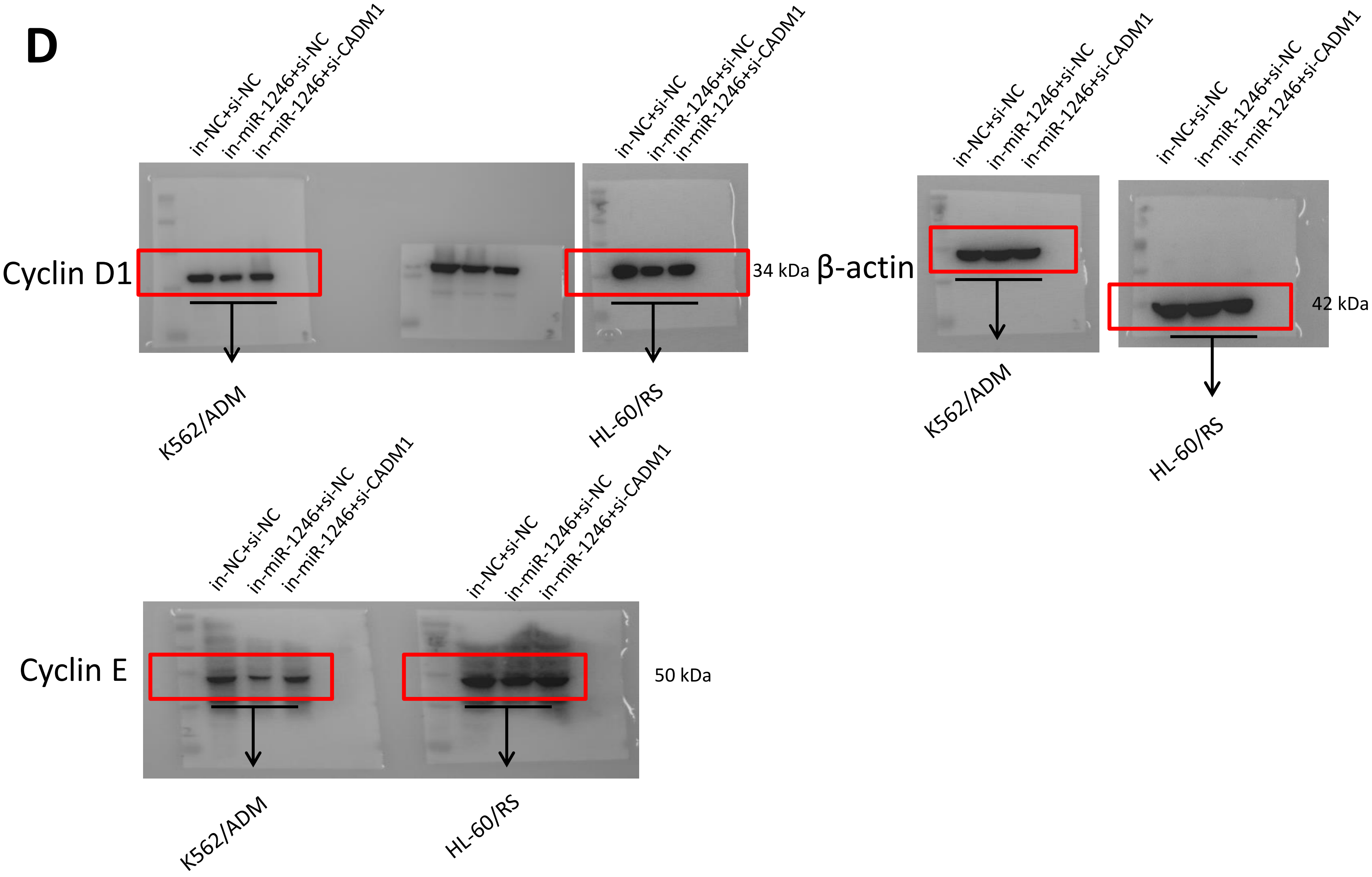
D
in-NC+si-NC
in-miR-1246+si-NC
in-miR-1246+si-CADM1
Cyclin D1
34 kDa
K562/ADM
HL-60/RS
β-actin
42 kDa
Cyclin E
50 kDa

Figure 4

E

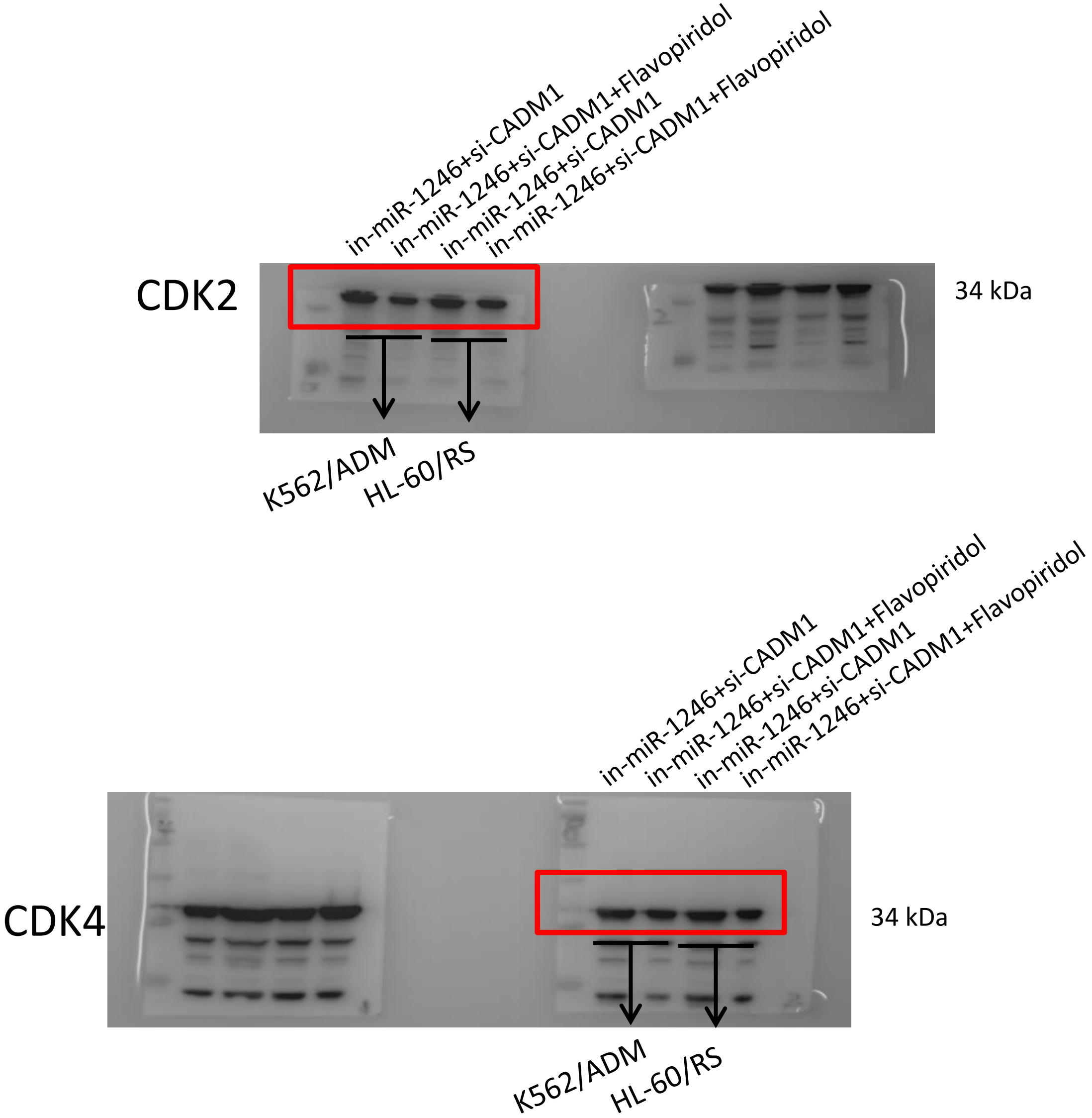
in-miR-1246+si-CADM1
in-miR-1246+si-CADM1+Flavopiridol
in-miR-1246+si-CADM1
in-miR-1246+si-CADM1+Flavopiridol
CDK2
34 kDa
K562/ADM
HL-60/RS
in-miR-1246+si-CADM1
in-miR-1246+si-CADM1+Flavopiridol
in-miR-1246+si-CADM1
in-miR-1246+si-CADM1+Flavopiridol
CDK4
34 kDa
K562/ADM
HL-60/RS

Figure 4

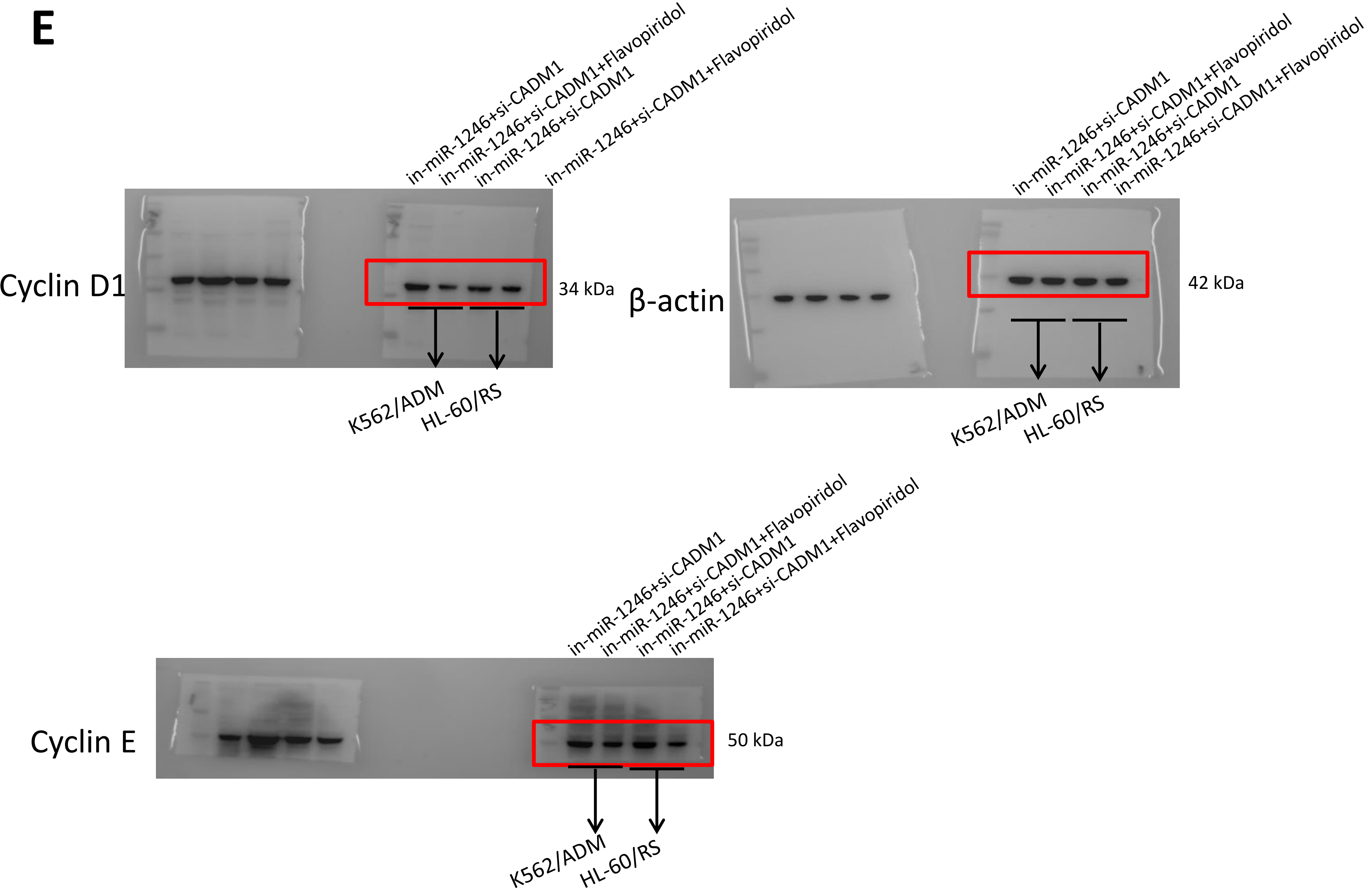
E
in-miR-1246+si-CADM1
in-miR-1246+si-CADM1+Flavopiridol
in-miR-1246+si-CADM1
in-miR-1246+si-CADM1+Flavopiridol
Cyclin D1
34 kDa
K562/ADM
HL-60/RS
in-miR-1246+si-CADM1
in-miR-1246+si-CADM1+Flavopiridol
in-miR-1246+si-CADM1
in-miR-1246+si-CADM1+Flavopiridol
β-actin
42 kDa
K562/ADM
HL-60/RS
in-miR-1246+si-CADM1
in-miR-1246+si-CADM1+Flavopiridol
in-miR-1246+si-CADM1
in-miR-1246+si-CADM1+Flavopiridol
Cyclin E
50 kDa
K562/ADM
HL-60/RS

Figure 4

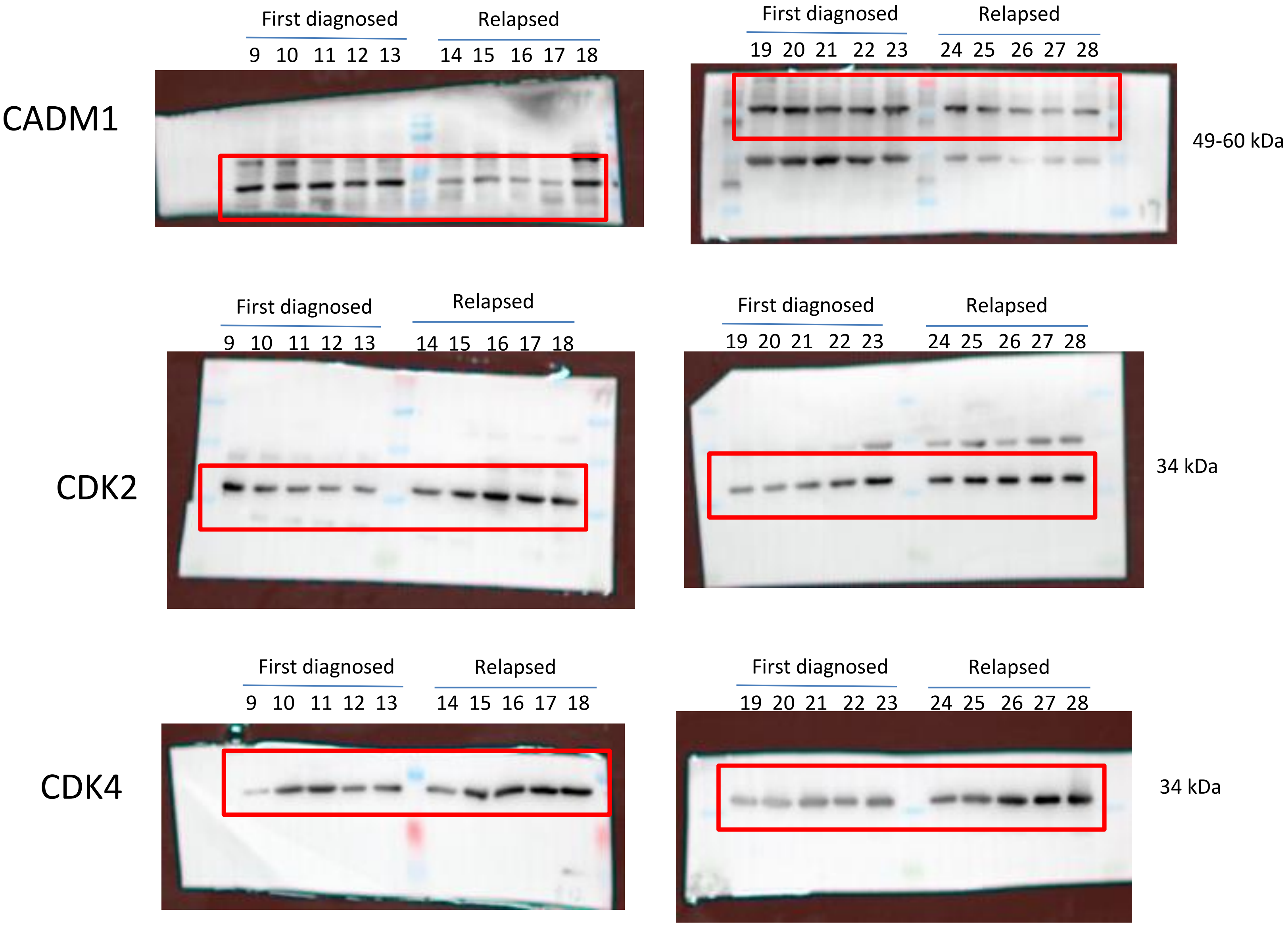

Supplementary Figure 1

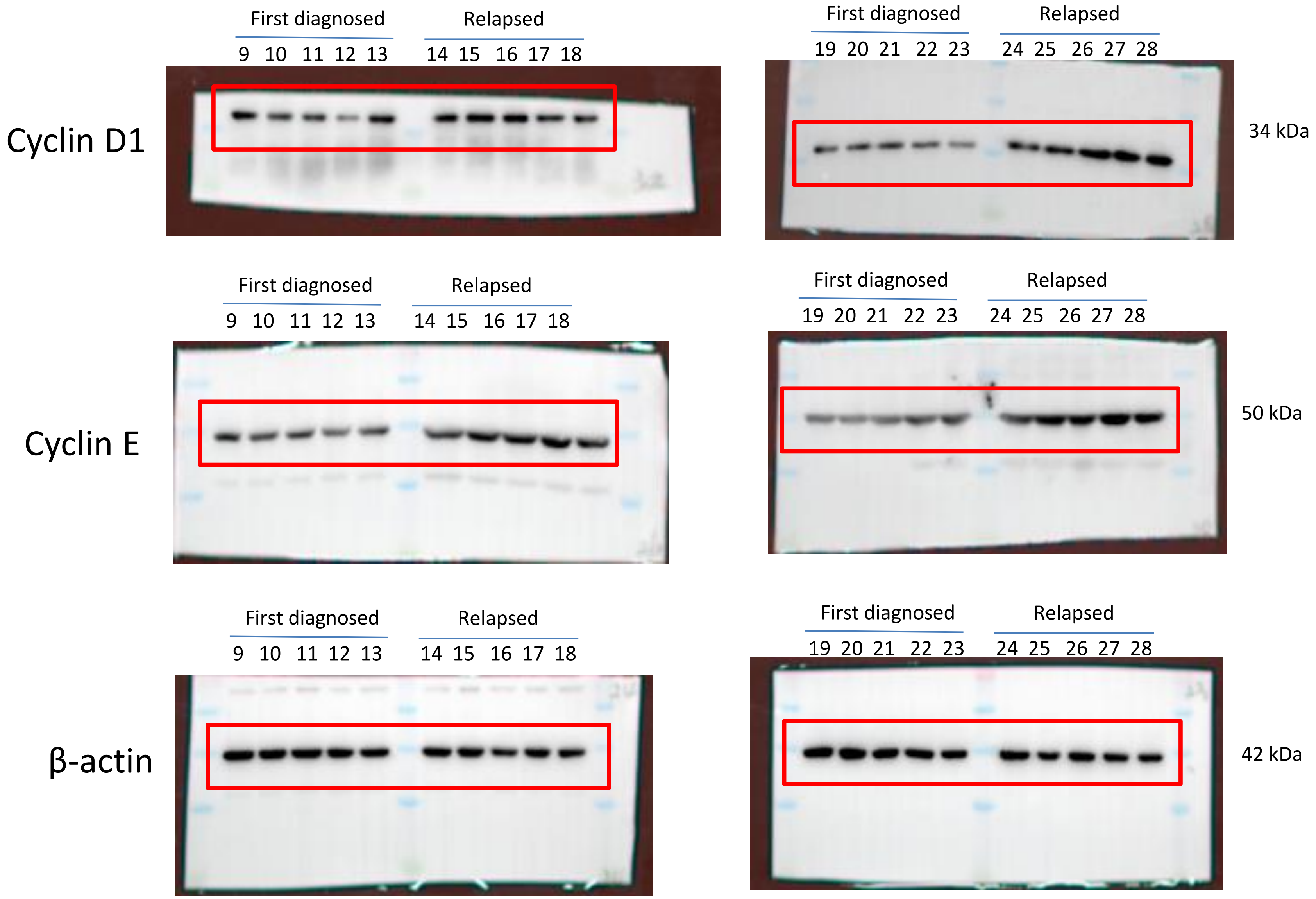

Supplementary Figure 1
