# Supplementary material for: CADM1 impairs the effect of miR-1246 on promoting cell cycle progression in chemo-resistant leukemia cells
Source: BMC Cancer. 2023 Oct 9;23:955. doi: 10.1186/s12885-023-11458-1 (PMC10561441; doi:10.1186/s12885-023-11458-1)
Supplement: Supplementary file 2 — Supplementary Material 2 [file 12885_2023_11458_MOESM2_ESM.docx]

**Supplementary file 1**

Table S1. The primer sequences for RT-qPCR. Table S2. The primer sequences for RT-qPCR. Table S3. The sequences of miR-1246 mimics, miR-1246 inhibitor and their controls. Table S4. The sequences of siRNAs of CADM1. Table S5. The primer sequences for luciferase reporter assay.

Table S1 The primer sequences for RT-qPCR

| Gene name |  | Sequences (5' to 3') |
| --- | --- | --- |
| CADM1 | Forward | ATACCGATCCCCCACAGGAA |
|  | Reverse | CTTCCACCTCCGATTTGCCT |
| β-actin | Forward | TGCTCCTCCTGAGCGCAAGTA |
|  | Reverse | CCACATCTGCTGGAAGGTGGA |

Table S2 The primer sequences for RT-qPCR

| miRNA name |  | Sequences (5' to 3') |
| --- | --- | --- |
| hsa-miR-1246 | Forward | TTCGACGTGAATGGATTTTTG |
|  | Reverse | TATCGTTGTACTCCAGACCAAGAC |
| U6 | Forward | CAGCACATATACTAAAATTGGAACG |
|  | Reverse | ACGAATTTGCGTGTCATCC |

Table S3 The sequences of miR-1246 mimics, miR-1246 inhibitor and their controls

| Name |  | Sequences (5' to 3') |
| --- | --- | --- |
| hsa-miR-1246 mimics | Sense | AAUGGAUUUUUGGAGCAGG |
|  | Antisense | UGCUCCAAAAAUCCAUUUU |
| mimics control | Sense | UUCUCCGAACGUGUCACGUTT |
|  | Antisense | ACGUGACACGUUCGGAGAATT |
| hsa-miR-1246 inhibitor | Sense | CCUGCUCCAAAAAUCCAUU |
| inhibitor control | Sense | CAGUACUUUUGUGUAGUACAA |

Table S4 The sequences of siRNAs of CADM1

| RNA name | sequence | |
| --- | --- | --- |
|  | sense（5'-3'） | antisense（5'-3'） |
| CADM1-Homo-1274 | GACGCAGACACAGCUAUAATT | UUAUAGCUGUGUCUGCGUCTT |
| CADM1-Homo-1074 | CCACCAUCCUUACCAUCAUTT | AUGAUGGUAAGGAUGGUGGTT |
| CADM1-Homo-718 | CCAGCGGUAUCUAGAAGUATT | UACUUCUAGAUACCGCUGGTT |
| NC | UUCUCCGAACGUGUCACGUTT | ACGUGACACGUUCGGAGAATT |

Table S5 The primer sequences for luciferase reporter assay

| Name | Sequences (5' to 3') |
| --- | --- |
| 3' UTR CADM1 sense | F: GTAATTCTAGTTGTTTAAACGAGCTCACTATTGTTACATTATGTGG |
|  | R: CCTGCAGGTCGACTCTAGACTCGAGCTATGGTCGGAATGGGTGCT |
| 3' UTR CADM1 antisense | F: CCTGCAGGTCGACTCTAGACTCGAGACTATTGTTACATTATGTGG |
|  | R: GTAATTCTAGTTGTTTAAACGAGCTCCTATGGTCGGAATGGGTGCT |

**Supplementary file 2**


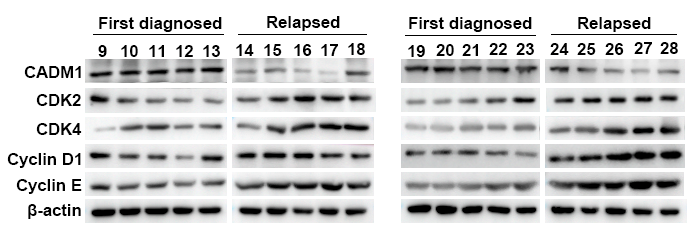


**Supplementary Fig. 1** The protein levels of CADM1, CDK2, CDK4, Cyclin D1 and Cyclin E were detected in samples from first diagnosed and relapsed patients.
